# Requirements for fast multianalyte detection and characterisation via electrochemical-assisted SERS in a reusable and easily manufactured flow cell

**DOI:** 10.1007/s00216-025-05763-w

**Published:** 2025-02-03

**Authors:** Maximilian E. Blaha, Anish Das, Detlev Belder

**Affiliations:** Institute for Analytical Chemistry, Leipzig University, Linnéstraße 3, 04103 Leipzig, Germany

**Keywords:** Surface-enhanced Raman spectroscopy, HPLC, Flow cell, Electrochemical SERS, SERS substrates

## Abstract

Surface-enhanced Raman spectroscopy (SERS) is a highly sensitive analytical technique that captures vibrational spectra of analytes adsorbed to rough coin metal surfaces with remarkable signal intensities. However, its wider application is limited by challenges in substrate range, quantification, and the disposable nature of SERS substrates partly due to irreversible analyte adsorption—commonly referred to as the ‘memory effect’. Overcoming these limitations and achieving real-time analysis in flow-through systems remains a key challenge for the advancement of SERS. This study presents a SERS flow cell incorporating an Ag-based SERS substrate and a Pt counter-electrode, enabling the investigation of how electrochemical methods can address existing challenges. Our approach demonstrates that signal intensities can be both enhanced and spectroelectrochemically modified. Additionally, the combination of constant solvent flow and electrochemical potentials enhances the longevity of the SERS substrate, facilitating multianalyte measurements while mitigating the memory effect. Key parameters have been systematically studied, including SERS substrate materials (silver and copper), solvents, buffers, supporting electrolytes, and electrochemical protocols. We achieved consistent and reproducible electrochemical tuning of SERS signals by using halogen-free electrolytes in polar solvents commonly used in techniques like HPLC. The versatility of the system was validated through the analysis of several model compounds and the sequential detection of multiple analytes. We also successfully applied the system to detect and characterise contaminants and pharmaceuticals, highlighting its potential for a wide range of analytical applications.

**Graphical Abstract:**

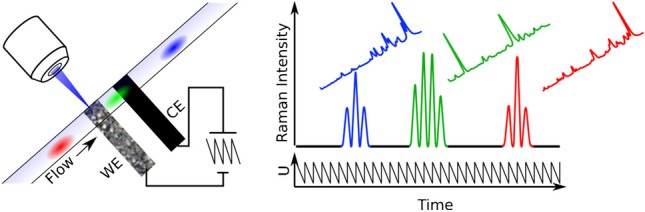

**Supplementary Information:**

The online version contains supplementary material available at 10.1007/s00216-025-05763-w.

## Introduction

Surface-enhanced Raman spectroscopy (SERS) is a promising technique that enables the detection of vibrational spectra of substances down to single molecular concentrations [1, 2]. The Raman signals of substances adsorbed to nanoparticles, rough surfaces, or nanostructured surfaces (SERS substrates) of mainly coin metals [1–3] are enhanced due to plasmonic interactions 10^3^ to 10^10^ fold [4–6]. Compared to classical Raman spectroscopy, whose most significant drawback is its low sensitivity, SERS signals appear in fluorescence-like intensities. SERS enables the label-free, noninvasive analysis of chemicals and biomolecules, allowing for the identification and quantification of their chemical composition through fingerprinting [7–10].

The potential of SERS has been shown in various applications such as environmental analytics, bioanalytics and biology, drug detection, food analytics, and material science [11]. Currently, the use of SERS in routine applications remains rare [12]. The main reasons herein rely upon the following. Although SERS can be used to quantify chemicals, the technique suffers from challenges regarding reproducible micromanufacturing of SERS-active surfaces, resulting in vast differences regarding the achieved signal enhancement [12–15]. Furthermore, the development of quantification and verification methods is extensive [13]. Regarding complex and biological samples, SERS is easily influenced by complex sample matrices [13]. SERS works best with molecules with functional groups that allow adsorption to coin metal surfaces such as thiols, carboxylic acids, and amines [16].

When SERS is combined with the simultaneous integration of electrochemical methods (EC-SERS), we enter the field of spectroelectrochemistry [5, 17, 18]. The application of spectroelectrochemistry offers additional features to the SERS measurements, such as surface activation, additional signal enhancement, and spectroscopic detectable alterations of the sample of interest which can be simultaneously investigated with the underlying redox reaction [5, 19]. The additional adsorption and desorption of the analyte, along with changes in its redox state on the SERS substrates, can be deliberately influenced to either make substances detectable or cause the signal to disappear. The latter is particularly relevant for reducing the so-called memory effect—a phenomenon where once-adsorbed molecules are difficult to remove, resulting in their signal persisting in subsequent measurements. This persistence hinders reliable multiple measurements on a single substrate, necessitating the use of a new substrate for each measurement. Furthermore, it impedes effective continuous-flow SERS detection, such as SERS detection in HPLC [20–23]. The integration of EC-SERS could be a tool to overcome the mentioned shortcomings [5].

In previous studies, a chip-based flow cell was developed to integrate an electrically contactable SERS substrate with a counter electrode [24]. The setup was primarily developed to overcome the memory effect and, thus, to regenerate the SERS substrate for subsequent measurements. However, the setup also allows the spectroelectrochemical properties of analytes to be investigated under micro flow conditions.

In this study, we have developed an advanced setup that includes potentiostats and an adapted chip design, enabling detailed studies to be carried out on the phenomenon of electrochemically modulated SERS signals in continuous flow.

To this end, we investigated different influential parameters, such as various SERS substrates and supporting electrolytes at multiple concentrations, solvent mixtures, and electrical potential programs. Furthermore, we demonstrated the usability and robustness of our system for several analytes and serial measurements. Besides optimising our approach to higher signal intensities, higher reliability, and faster electrochemical processing, we also explore its limitations.

## Materials and methods

### Chemicals

Copper and silver wires (diameter 0.25 mm, 99.9%) were purchased from Sigma-Aldrich Chemie GmbH, Steinheim, Germany. Gold and platinum wires (diameter 0.25 mm, 99.9%) were purchased from Alfa Aesar GmbH, Karlsruhe, Germany. Analytes were purchased as follows: methylene blue (Reachim), crystal violet (Sigma-Aldrich), malachite green (Kallies Feinchemie KG, Sebnitz, Germany), adenine (Fisher Scientific GmbH, Schwerte, Germany), cytosine (Sigma-Aldrich), L-Dopa (Sigma-Aldrich), melamine (Fisher Scientific GmbH), and guaifenesin (Alfa Aesar). Electrolytes: PBS (Fisher), NH_4_OAc (Merck KGaA, Darmstadt, Germany), NaOAc (Merck), KNO_3_ (VEB Laborchemie Apolda, Apolda, GDR), Bu_4_NOAc (Alfa Aesar), NaCl (Carl Roth GmbH & Co. KG, Karlsruhe, Germany), Bu_4_HSO_4_ (Sigma-Aldrich), NaCitrate (Isocommerz GmbH, Berlin, Germany). Reagents were purchased as follows: NaOH (Carl Roth GmbH), NH_3_ 35% (Fisher), HNO_3_ 65% (Merck), HAuCl_4_ (Fisher), Cu(OAc)_2_ (VEB Laborchemie Apolda), AgNO_3_ (Roth), MeOH (HiPerSolv Chromanorm, VWR, Darmstadt, Germany), MeCN (VWR, Darmstadt, Germany), H_2_O (desalted).

### SERS substrate manufacturing

As in our previous study [24], we used an etched silver wire adapted from Wijesuriya. Burugapalli et al. [25] manufactured as follows: the silver wire was flattened using a mechanical press (PO10H; Paul Otto Weber; Germany) for 120 min at 30 kN. The silver wire was ultrasonicated for 3 min in MeOH for cleaning. Etching was performed by immersing the flattened wire for 30 s in 35% NH_3,_ followed by etching in 6 M HNO_3_ for 10 s. SERS substrate manufacturing for other SERS substrates is described in the SI Chapter 1.

### Experimental setup

**Fig. 1:**
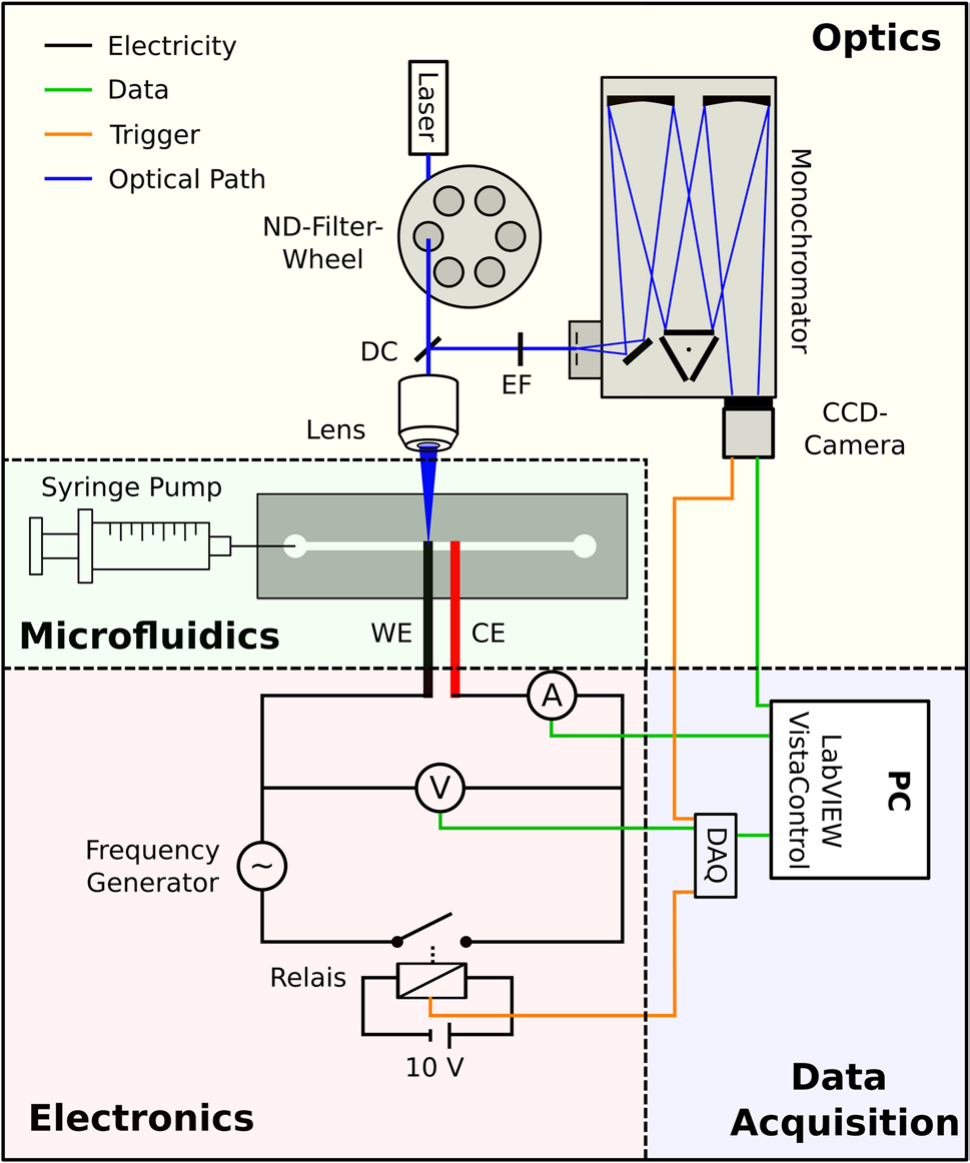
Experimental setup

The experimental setup can be divided into a microfluidics part, an optical part, an electronics part, and a data acquisition part. The interaction of these parts is shown in Fig. 1. Regarding the optical part, two different Raman systems with different excitation wavelengths of 473 nm and 532 nm were used, as described in the related parts. The experimental section describes the setups in their most common configurations. Variances from the described settings are described in the results part.

### Microfluidics

The microfluidics part is based on our foil chips manufactured as described in the related chapter. It involves a SERS-active wire called working electrode (WE) from an electrochemical perspective. If not described further, a flattened and etched Ag-wire was used. A flattened Pt-wire was used for the counter electrode (CE). The chip was constantly flushed at 200 µL/min with different buffers and analytes using a PHD 2000 syringe pump (Harvard Apparatus, USA) equipped with 10-ml high precision glass syringes (CETONI, Germany). If not stated otherwise, all experiments were carried out with a constant flow of 200 µL/min. The liquid was transferred to the chip using Teflon tubes (1.58 mm OD; 0.3 mm ID; Sigma-Aldrich, USA) and Luer lock adapters. In our experiments’ later stages, we sampled model compounds and analytes in our micro-SERS-Sensor using a Hexaport injection valve (Rheodyne 7725i, Rheodyne, USA).

### 473 nm optical setup

For optical detection and Raman signal acquisition, the chip was placed on an IX 71 inverted microscope (Olympus, Japan) equipped with a LUCPlanFl 40 × objective (NA 0.6; Olympus, Japan). The optical setup is based on a confocal modular Raman measurement setup (S&I Spectroscopy & Imaging GmbH, Germany) and incorporates the following components. A 473 nm Cobolt Blues 50 mW laser (Cobolt AB, Sweden) was used for excitation. The excitation light passed a neutral density filter wheel and a dichroic mirror (DC) to be transferred on the SERS substrate. The scattered light was collected with the same lens, passed an edge filter (EF), and was fed into a spectrograph/monochromator (Acton SP2750, Princeton Instruments, USA) with an entrance slit of 150 µm and a grating 600 lines/mm. For detection, a Peltier cooled 1600 × 200 CCD Camera (ProEM 1600 × 200 CCD Camera, Princeton Instruments, USA). For excitation, a laser power of 2.5 mW was used if not stated otherwise.

### 532 nm optical setup

In our 532 nm Raman Signal acquisition system, the chip was placed on an IX 73 inverted microscope (Olympus, Japan) equipped with a LUCPlanFl 40 × objective (NA 0.6; Olympus, Japan). The optical setup is based on a confocal modular Raman measurement setup (S&I Spectroscopy & Imaging GmbH, Germany) and incorporates the following components. A 532 nm Cobolt Samba laser (100 mW, Cobolt AB, Sweden) was used for excitation. The excitation light passed a neutral density filter wheel and a dichroic mirror (DC) to be transferred on the SERS substrate. The scattered light was collected with the same lens, passed an edge filter (EF), and fed into a spectrograph/monochromator (SpectraPro HRS-500, Princeton Instruments, USA) with a 1200 lines/mm grating. A Peltier-cooled 1600 × 250 CCD Camera (DR-324-FI-RES, Andor, UK) was used for detection.

### Electronics

We incorporated a signal generator (Rigol Technologies, DG 1032 dual-channel, China) or a Potentiostat (PGSTAT10, Eco Chemie BV, The Netherlands) in a 2-electrode configuration in order to perform online electrochemistry. Only negative voltages were applied to the working electrodes to prevent corrosion. Voltage was measured in real time using the analogue input of a USB-6000 (National Instruments, USA) as a data acquisition (DAQ) system. The current was measured using a precision multimetre (DMM 6500, Keithley, Germany). A triggered relay (HLRELM-4, Sertronics GmbH, Germany) was used to switch the electrical circuit on and off.

### Data acquisition

For data acquisition, we used a PC running LabVIEW 2017 (National Instruments, USA) for collecting the input data of the multimetres and VistaControl V4.2 Build 12596 (S&I Spectroscopy & Imaging GmbH, Germany) for collecting the output of the CCD Camera. The LabVIEW script also triggered the CCD Camera and the relay, allowing the synchronisation of the data collection of the CCD Camera and the multimetres.

### Chip design, fabrication, and assembly

**Fig. 2:**
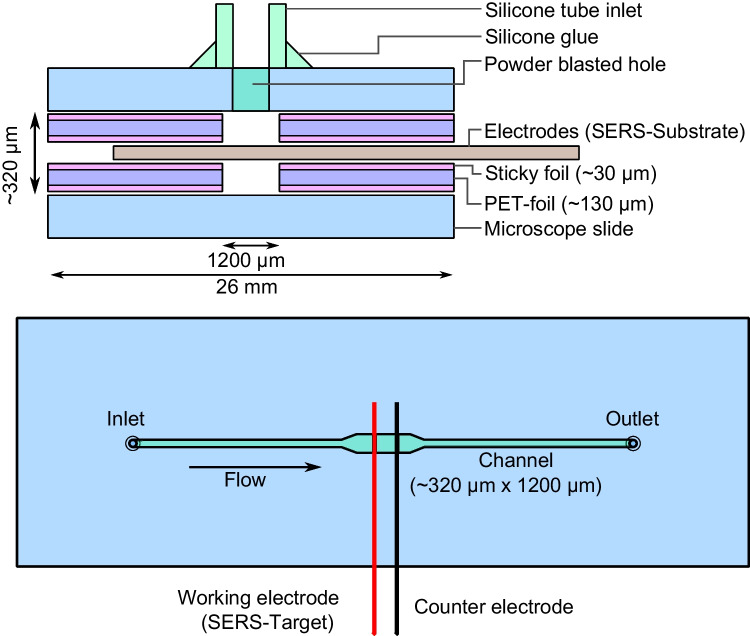
Schematic sketch of the used chip design

Chip fabrication was optimised to produce chips reproducibly and quickly on our foil chip basis, which was established in a prior study [24]. A sketch of the used chip design is displayed in Fig. 2. The chips were manufactured as follows: A PET-foil (Melinex T506; König GmbH Kunststoffprodukte, Germany) was covered on the upper and the lower side with 60 µm (~ 30 µm after pressing) 467MP Adhesive foil (3 M, Germany). A laser cutter (VLS 2.30; Universal Laser Systems, Austria) was used to cut channels in the foil system. The prepared foils were placed on microscope slides (76 × 26 × 1 mm, ‘Menzel-Gläser’, Thermo Scientific, Germany). For the counter electrode, we used pressed Pt wires using a mechanical press (PO10H; Paul Otto Weber; Germany) for 120 min at 30 kN. The flattened counter electrode and the SERS substrate were placed ~ 1 mm apart on the sticky side of one of the prepared glass slides. In another prepared glass slide, holes were powder blasted (Sandstrahler Point II, Barth Serienapparate, Germany) for the chip’s inlet and outlet. Both glass slides were then put on top of one another. Both glass slides with incorporated electrodes were pressed using a mechanical press (PO10H; Paul Otto Weber; Germany) for 30 min at 5 kN. Inlets were glued on the inlet and outlet holes of the chip using ~ 1 cm long pieces of silicone tube (3.8 mm OD; 1.3 mm ID; Fischer Analytics GmbH, Germany) and silicone glue (Elastosil E43, Wacker Chemie, Germany). After pressing, we ended up with a channel height of ~ 380 µm and used a channel width of 800 µm.

### Enhancement-factor calculations

Since we focused on utilising SERS for analytical applications, we employed the analytical enhancement factor [26]. $AEF$ is calculated as shown in formula 1.

1 $$AE{F}_{1178 c{m}^{-1}}=\frac{{I}_{SERS}}{{I}_{RS}}\bullet \frac{{c}_{RS}}{{c}_{SERS}}$$

For the intensities, the peak height over the background was used after background correction. ${I}_{SERS}$ referring to the SERS intensity at a specific wavenumber, ${I}_{RS}$ referring to the normal Raman intensity at a specific wavenumber, ${c}_{RS}$ referring to the concentration of the analyte used in non-enhanced Raman spectroscopy, and ${c}_{SERS}$ referring to the concentration of CV used for SERS measurements. Crystal violet (CV) (10 µM) was used to test the enhancement factor, and the intensities at a wavenumber of 1178 cm^−1^ were compared [24].

## Results and discussion

The first studies on the regeneration of SERS substrates in micro-flow cells used electrochemically very simple and not well-defined setups with very high clamp voltages of up to 100 V [21, 24]. In order to investigate this so far barely understood effect named electrical regeneration of SERS substrates, we have developed a system that allows the application of defined potentials to SERS substrates in a micro-flow setup. As a starting point, we used a modified version of a chip described in a previous work [24]. But instead of using a simple DC laboratory power supply to apply the clamp voltage, we used a frequency generator for time-controlled voltage applications as well as a potentiostat. In both configurations, we worked with a 2-electrode system to maintain simplicity. In the process of electrochemical alteration, it is crucial to prevent the total electrolysis of the solvent by avoiding the formation of disruptive gas bubbles that could interfere with the system and the detection of the model analyte. We achieved the most consistent results when applying potentials down to − 3.5 V. Negative potentials were exclusively used on the SERS substrate, as positive potentials can induce corrosion of the Ag-SERS substrate leading to signal loss. Therefore, to facilitate electrochemical improvements, a triangular periodic wave function was applied within the range of 0 to − 3.5 V, with a time period of 70 s, resulting in a scan rate of 0.1 V/s.

**Fig. 3:**
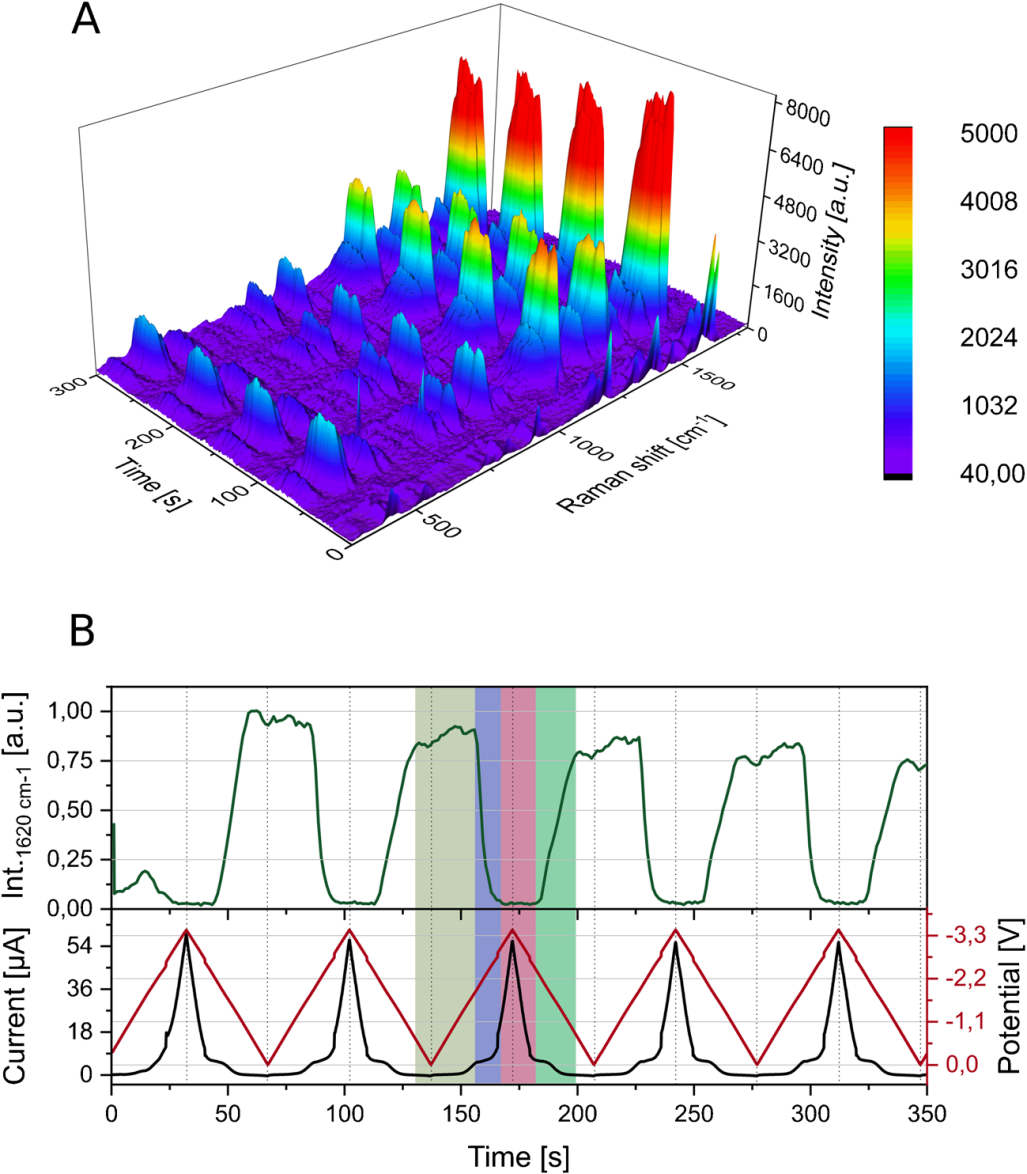
Intensity of the Raman signal of 10 µM CV in 66 mM PBS at pH 7 Period duration 70 s, Potential 0 V to − 3.5 V. Analyte was flushed in constant flow: 200 µl/min. (473 nm; 2.5 mW; 600 lines/mm; 40 × objective; 1 s integration time) **A** Intensity at 1620 cm^−1^ in dependency of the applied potential. **B** 3D plot of the Raman spectrum of CV over time

Using these initial settings, we recorded the data displayed in Fig. 3. Figure 3A contains the whole Raman spectrum of CV which evolves over time for periodic SERS detection. A periodic signal loss can be seen due to the reduction of the analyte into a form that produces weaker SERS signals. Also, the signal regrowth over time can be observed, illustrating that the SERS substrate is stable for multiple reduction cycles. Figure 3B depicts the time-dependent signal intensity progression for the most prominent SERS peak of CV (1620 cm^−1^). The progression of the applied potential and the measured current are displayed in Fig. 3. The minima and maxima of the potential curve are marked on the intensity line to allow for an assignment of signal intensity and potential. The highlighted section in Fig. 3B provides a better understanding at which exact potentials the signal intensity starts to change. It can be observed that the signal stays constant until a potential of − 1.9 V (highlighted in olive). Subsequently, the reduction of CV takes place, leading to a gradual decrease in signal intensity (highlighted in blue) until the signal completely disappears at − 2.98 (highlighted in purple). In the backsweep, the signal starts to regrow at − 2.25 V and reaches its maximum intensity at − 1.00 V (highlighted in green) indicating a reoxidation of CV adsorbed to the SERS substrate. The described results can be considered as a starting point for our investigation, showing how the changes in various parameters will further influence the signal evolvement of CV, focusing on system stability and high intensities and testing the system to determine whether it is broadly applicable. We optimised for universal measurement conditions to enable the detection of multiple analytes in online techniques such as HPLC.

### Variation of the supporting electrolyte

The choice of supporting electrolyte is an important parameter in electrochemical processes. In the context of this study, it must meet the dual criteria of serving as a conductive medium and being compatible with SERS. It should not interfere negatively with the SERS substrate and should not hinder the adsorption of analytes to the substrates. Also, the electrolyte must satisfy specific requirements, including solubility in the chosen solvent inertness under the experimental conditions [27, 28]. For future broader applications, such as SERS detection in chip-HPLC, the pH of the supporting electrolytes is also important. Hence, these parameters can potentially significantly impact the signal quality. Therefore, in another set of experiments, we studied the influence of different supporting electrolytes and buffers (commonly used for HPLC) and compared their performance regarding the signal progression of the model analyte. For this purpose, we chose concentrations of 100 mM for each electrolyte used. All the other experimental conditions were used as in the previous section. SERS detection was realised using a 473 nm laser with a power of 2.5 mW and an integration time of 1.0 s. The electrolyte’s pH values and conductivities are listed in Table S1 in the SI.

**Fig. 4:**
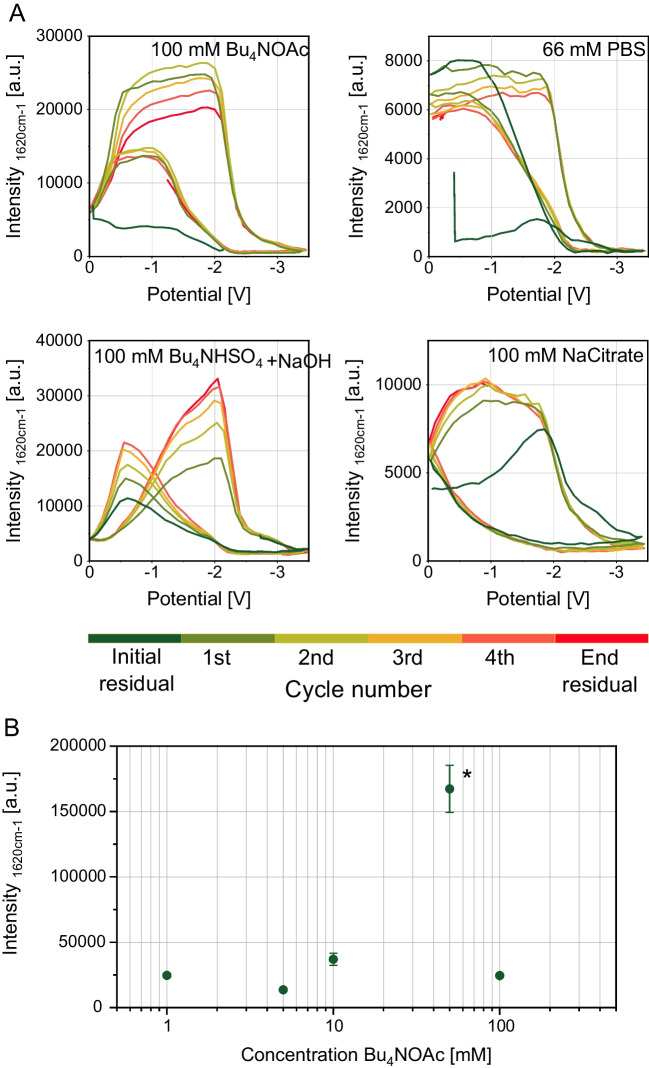
Power source: frequency generator with triangular potential between 0.0 and − 3.5 V and a period duration of 70 s. Measurement parameters: 473 nm; 2.5 mW; 600 lines/mm; objective: 40-fold; integration time: 1 s; flow: 200 µL/min; indicational band: 1620 cm^−1^. **A** Influence of different supporting electrolytes on the progression of the SERS-Intensity of 10 µM CV in aqueous solution. **B** Influence of the concentration of Bu_4_NOAc on the SERS-Intensity of 10 µM CV in aqueous solution. With * marked measurement was recorded with 0.25 mW laser power and signal intensity corrected by multiplying tenfold

It can be seen from Fig. 4A that the reduction process can be studied independently from the electrolyte used. However, significant differences in the signal intensity curves were observed. In the case of PBS, the signal intensity remained stable until the electrical reduction began at − 1.9 V. In the case of Bu_4_NOAc and Bu_4_NHSO_4_, the signal intensity initially increased, reaching a maximum at an applied potential of − 2 V. Reaching potentials below − 2 V, the signal starts to perish. In the down sweep, the intensity maximum is not reached. Regarding sodium citrate, the signal intensity reaches its maximum at − 1 V during the downsweep and starts to perish rapidly below − 1.9 V. During the backsweep, no signal maximum is reached. This is likely due to the competition between cations in the solution for adsorption sites on the SERS substrate. In all electrolyte experiments, the intensity during the upsweep is consistently lower than during the downsweep. We hypothesise that this difference in intensity curves is influenced by the behaviour of the electrochemical double layer. The Raman intensity of CV is measured on the negatively charged electrode, which, therefore, attracts cations. To this end, the relevant cations in the solutions are CV^+^ as analyte, H^+^, and dependent on the used electrolyte K^+^, Na^+^, or Bu_4_N^+^. The signal intensity primarily depends on the extent of CV^+^ adsorption on the surface relative to other cations. Signal enhancement occurs when CV^+^ is preferentially attracted to the negatively charged Ag surface over other cations in the solution. We attribute the signal loss to the reduction of CV^+^ ions. KNO_3_, NaOAc, and NH_4_OAc were also tested, and the results were similar to those of PBS. A detailed discussion is given in Figure S8 in the SI. Electrolyte solutions containing high concentrations of halides were not usable since we experienced an almost complete signal loss over time. We explain this signal loss due to interactions of the halide ions with the roughened silver surface under the influence of laser light.

In our further study, we used Bu_4_NOAc. Next to a stable SERS intensity over time, we achieved a stable reduction CV from the surface over multiple cycles and a signal enhancement at potentials between 0 and − 2 V. Furthermore, it is soluble in polar organic solvents such as MeOH and MeCN.

### Concentration of the supporting electrolyte

After finding that Bu_4_NOAc is an excellent supporting electrolyte capable of providing additional enhancement and being solvable in organic solvents, we adjusted the concentration necessary to achieve maximum enhancement. Besides the concentration of Bu_4_NOAc, all experimental parameters stayed the same as before. We tested the following concentrations: 1 mM, 5 mM, 10 mM, 50 mM, and 100 mM. Figure 4B displays the achieved maximum intensities for each concentration. The error bars display the fluctuations during the measurement. With 1 mM and 5 mM concentrations, no complete reduction of CV was achieved at − 3.5 V due to low conductivity. At 50 mM concentration, we used only 10% of the laser power (0.25 W) used for the other concentration due to vast detector oversaturation. The yielding intensity was normalised tenfold to become comparable to the other intensities. As shown in Fig. 4B, 50 mM Bu₄NOAc produced the highest intensities, so it was chosen for all subsequent experiments.

### Varying the solvent mixture

**Fig. 5:**
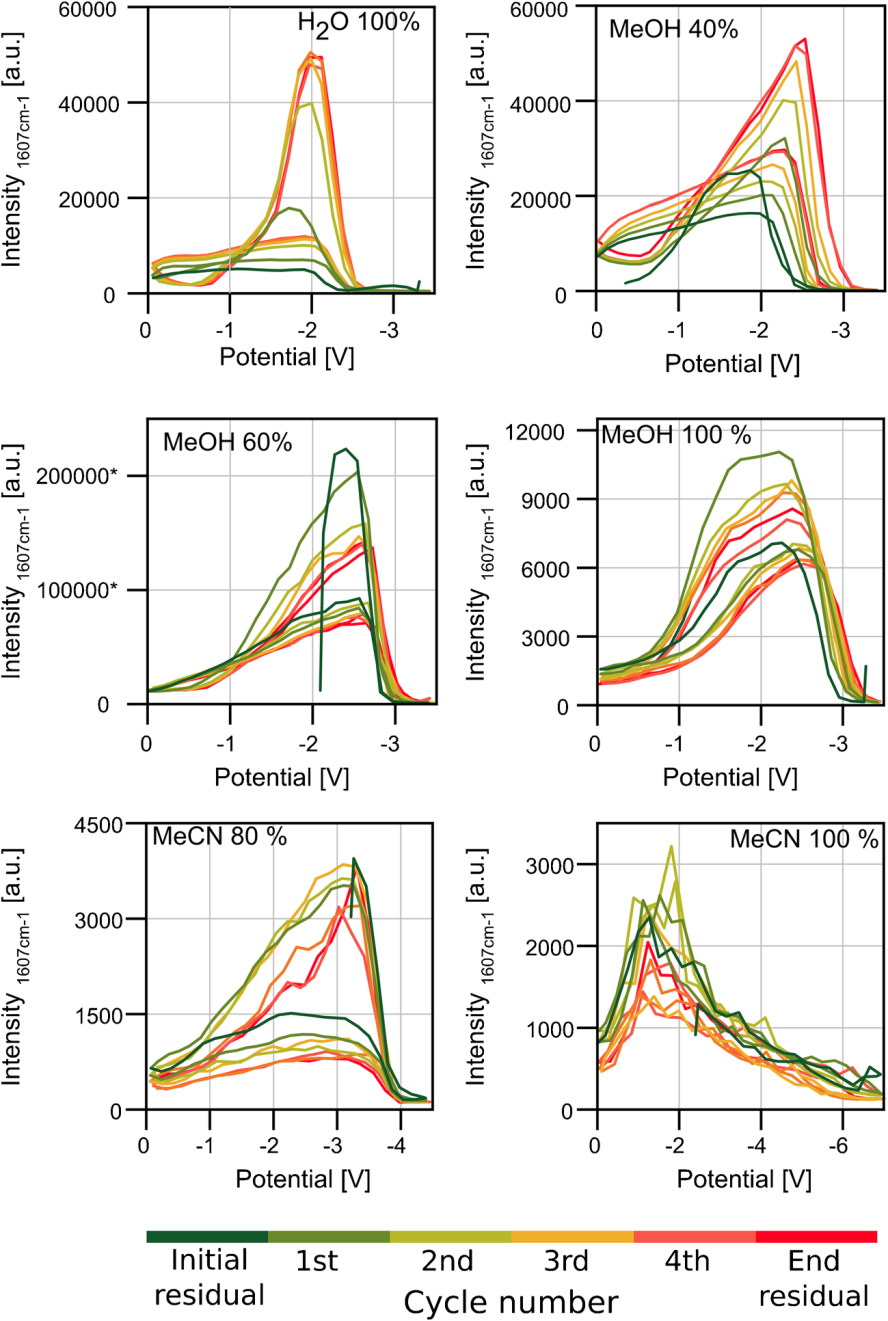
Intensity curve of 10 µM CV dissolved in different solvent mixtures containing 50 mM Bu_4_NOAc. Power source: frequency generator with triangular potential between 0.0 and − 3.5 V and a period duration of 70 s. Measurement parameters: 532 nm; 4.1 mW; 600 lines/mm; objective: 40-fold; integration time: 1 s; flow: 200 µL/min; indicational band: as indicated

In coupling SERS with electrochemical methods and online techniques, the solvent must also be compatible with all the techniques used. Using a solvent that supports the analyte’s adsorption to the surface favours SERS. In the current study, we used polar solvents such as H_2_O, MeCN, and MeOH, which are compatible with the chip material. From the standpoint of online techniques, these solvents mimic those usually used in RP-HPLC. From an electrochemical point of view, the solvent has to dissolve both the supporting electrolyte and the analyte while being inert to the potentials applied. We aim to investigate the compatibility of the repeatable spectroelectrochemical use of the SERS substrate with different solvent mixtures while optimising the solvent mixture and demonstrating limitations. To investigate the influence of solvent mixtures on the overall functionality of our system, we started with an aqueous solution as used so far and gradually enhanced the fraction of MeCN and MeOH in 20% steps. For detection, we used our 532 nm optical system. The most significant intensity curves of the measurement are displayed in Fig. 5. The other datasets are shown in Figure S10 in the SI. Overall, we noticed that the spectroelectrochemical alteration stayed fully functional in mixtures of MeOH and H_2_O in each ratio. We achieved excellent signal intensities with H_2_O/MeOH mixtures containing up to 80% MeOH. Regarding MeOH solutions, the potential necessary to reach a reduction of the CV slightly shifted to higher potentials with higher fractions of MeOH. With aqueous solutions, we reached the highest intensity at a potential of − 2.0 V and a full reduction at − 2.4 V. When using 100% MeOH, the highest intensity was reached at a potential of − 2.4 V, and a complete reduction was achieved using − 3.4 V.

In mixtures containing MeCN, the achieved intensities were lower in general. When using mixtures with H_2_O and MeCN, the difference in the reduction potential is even more significant. With MeCN fractions of up to 60%, the overall behaviour of our system stayed stable, and we could stay within the preset parameters. In a mixture containing 80% MeCN, we achieved the highest signal intensity at −3.5 V and a complete reduction at −3.9 V, lowering the potential to − 4.5 V. Although we lowered the potential, we experienced no disturbing gas bubbles due to the degradation of the solvent mixture. Regarding pure MeCN, a slow decolourisation of CV in the solution was visible. Higher potential differences were necessary to achieve a complete intensity loss of CV on the electrode. To reach minimal intensity, at least − 6.0 V had to be applied. Maximum intensity was achieved around − 2.0 V. Overall, our model system appeared unstable when using pure MeCN as solvent. The signal displayed in Fig. 5 is unstable, as seen by the rough shape of the intensity curve. Overall, we consider the spectroscopic observation of the reduction of CV fully functional and independent of the solvent mixture, but the reductive potential has to be adjusted. Comparing MeOH and MeCN solvent mixtures, MeCN altered the behaviour of our model system more significantly than MeOH and gave a significant background spectrum, especially when using high fractions of MeCN. We used MeOH/H_2_O 50/50 for further optimisations since it gave us the best combination of intensity and stability. Fractions of methanol lowered the solubility of gases in the solution, enhancing system reliability.

### Varying the SERS substrate material

**Fig. 6:**
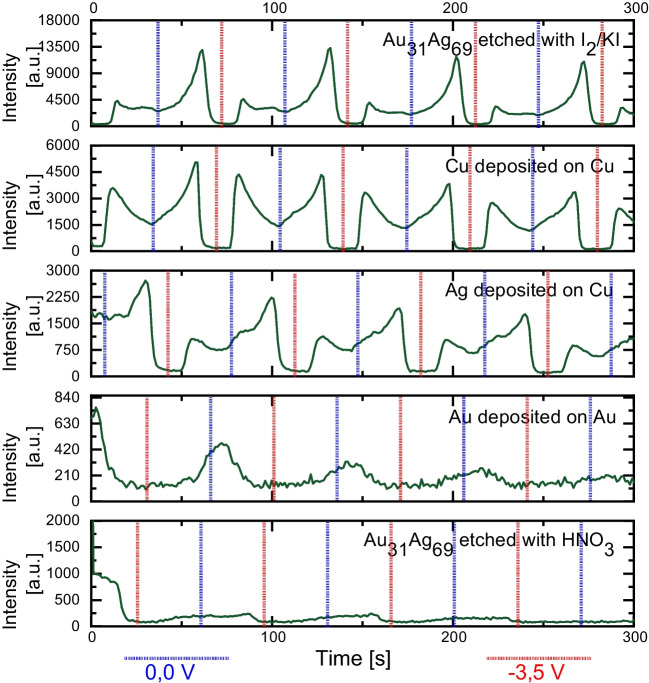
Progression of the SERS signal of 10 µM CV on different substrate materials, while a triangular potential (0.0 to − 3.5 V; period duration: 70 s) is applied. Experimental parameters: Excitation wavelength: 473 nm; laser power: 2.5 mW; integration time: 1000 ms; flow: 200 µL/min; indicational band: 1620 cm^−1^

An important aspect of obtaining a sensitive SERS signal and stable detection is selecting an appropriate material for the SERS substrate. The choice of the plasmonic material for the SERS substrate is crucial since it influences the spectra, the detected intensities and the excitation wavelength needed. Ag-SERS substrates are most effective when excited within the blue wavelength range of the electromagnetic spectra. The low wavelength results in higher SERS intensities but might sometimes interfere with fluorescence. One drawback of Ag substrates is their susceptibility to corrosion. On the other hand, Au works best in the red spectrum range. Although it often gives lower intensities, it is much more stable against oxidation. Cu substrates also work best using higher excitation wavelengths but suffer from low enhancement factors and corrosion, so Cu is rarely used [11, 29]. We investigated if there are limitations to the substrate material from the spectroelectrochemical point of view and how to improve it for online applications. This was quite challenging because the SERS substrate had to be contacted by electrical circuits. Conversely, the SERS substrate should also be stable to multiple electrical cycles with prolonged exposure to the laser. Therefore, we used different strategies to manufacture SERS substrates of various materials and material compositions. For manufacturing, we used methods such as etching and electrical deposition as follows: (1) a metal wire consisting of Au_31_Ag_69_ was etched with I_2_/KI, (2) Cu was electrochemically deposited on a Cu wire, (3) Ag nanostructures were grown on Cu via electroless deposition, (4) Au was electrochemically deposited on an Au-wire, and (5) Au_31_Ag_69_ was unalloyed using 65% HNO_3_. The exact protocols for each substrate are noted in the SI in Chapter 1. We used the experimental parameters as optimised so far to ensure that the system remained functional, while the SERS substrates were replaced. We measured the evolvement of continuously flowing CV-SERS signals dependent on the applied potential. To stay comparable, we stuck with an excitation wavelength of 473 nm, although not optimal for Cu and Au-based substrates. The resulting intensity curves of all substrates are shown in Fig. 6. Note that none of the protocols was optimised for high enhancement factors. The intensity curves are to be discussed as follows: the Au_31_Ag_69_ etched with I_2_/KI: I_2_/KI is aggressive to both Au and Ag. We consider the surface to consist of a mixture of Au and Ag. We experienced an increased enhancement factor of 1.8 × 10^4^ together with a stable reduction of crystal violet. Next, we achieved an enhancement factor of up to 3.5 × 10^3^ for the Cu deposited on the Cu substrate, which was sufficient for our experiments. Although the EF here was lower than the Au_31_Ag_69_ etched with I_2_/KI, the reduction process together with the signal enhancement worked perfectly for our experimental condition, as evident in Fig. 6. The achieved EF was 1.8 × 10^3^ for Ag deposited on Cu substrate.

An EF of 1.0 × 10^3^ was obtained for the Au deposited on the Au substrate. However, besides a low intensity, a fast, and permanent loss in signal intensity was observed making this substrate unusable for our purpose. Finally, the Au_31_Ag_69_ wire unalloyed with 65% HNO_3_ showed the same characteristics as the Au substrate, although delivering an EF of 7.3 × 10^3^. HNO_3_ selectively removes Ag from the alloy. The resulting elemental composition was determined to be Au_75_Ag_25_. Therefore, we can conclude that Au-based substrates are incompatible with our measurement conditions. Potential mechanisms of signal loss due to the laser power are microheating and sintering, which alter the nanostructure of the surface or cause analyte decomposition [30]. Hence, Cu- and Ag-based substrates are fully functional within our setup. We however experienced some instabilities with mixed surface materials.

### Trimming the voltage function towards fast spectroelectrochemical detection

**Fig. 7:**
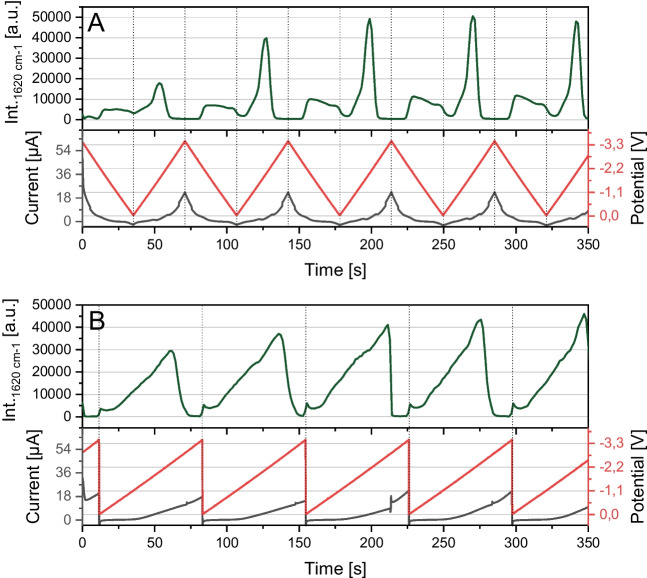
Intensity curves of the SERS-Spectra of 10 µM CV dissolved in 50/50 MeOH/H_2_O influenced by different functions of applied potential. Current and potential are indicated. A potential between 0.0 and −3.5 V was used with a period duration of 70 s. Measurement parameters: (532 nm; 4.1 mW; 600lines/mm; objective: 40-fold; integration time: 100 ms; flow: 200 µL/min; indicational band:1607 cm^−1^). Top: Triangular Potential. Bottom: Sawtooth-potential

**Fig. 8:**
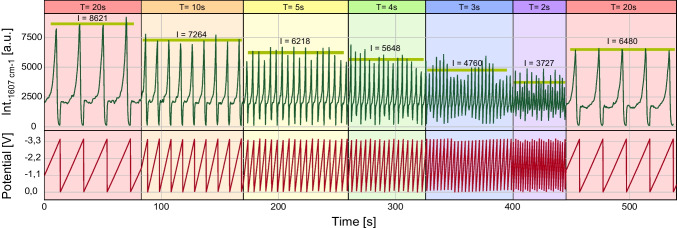
Intensity curve of CV in a microfluidic SERS sensor influenced by alternating frequency of a sawtooth voltage potential. A frequency generator was used as power source with a sawtooth potential varying between 0.0 and − 3.5 V. The period duration was gradually lowered. 10 µM CV dissolved in MeOH/H_2_O 50/50 containing 50 mM Bu_4_NOAc were flushed in a constant flow through the chip. (532 nm; 8.1 mW; 600 lines/mm; objective: 40-fold; integration time: 100 ms; flow: 200 µL/min; indicational band:1607 cm^−1^)

For real-time analysis in continuous flow applications, it is crucial that the analytes are analysed quickly and at a high sampling rate. For real-time application, the analyte must be adsorbed to the SERS substrate, the spectrum has to be recorded and saved, and the analyte has to be stripped from the surface to avoid carry over effects. Additionally, the SERS substrate has to be depolarised to establish the conditions for adsorption in the next cycle. As visible in the intensity curves of Fig. 4 and Fig. 7A, the intensity of CV is usually lower during the upsweep (backsweep) than during the downsweep. We consider the upsweep unnecessary to speed up the electrochemical cycling process since it gives lower intensities. In one first optimisation step, we recorded the spectrum without a downsweep, as shown in Fig. 7, resulting in a sawtooth-shaped waveform. The dotted lines indicate the extreme points of the potential and the related intensity. In the triangular-shaped potential, we see the local minimum of the potential at 0 V and the absence of a signal at − 3.5 V. After understanding the influence of a sawtooth-shaped potential on the intensity curve, the next step is to speed up the process to evaluate the limits of our system so far. To record SERS spectra fast, we lowered the exposure time of the sensor down to 100 ms. It has to be considered that the camera used for recording the spectra needs around ~ 300–500 ms to process and save the data after a spectrum was recorded. This time required for transferring and saving the data to the computer is not predictable and is to be seen as a random number in the given range. Furthermore, this saving time is the bottleneck of our system’s measuring speed. On average, we could record one spectrum per ~ 500 ms. We lowered the period duration of the sawtooth-shaped potential step by step, starting with a period duration of 20 s. The resulting intensity curve is shown in Fig. 8. Displayed is the course of the intensity over time, while the period duration of the potential is lowered step by step. The cycle duration used in every part of the diagram is indicated above. We were able to reduce the period duration down to 2 s. Lower period durations were not practical in the context of acquisition times of around 500 ms. It is visible that with lower period durations, the maximum achievable intensity decreases. To ensure that the intensity loss is due to the used period duration and not a decrease of the SERS substrate’s enhancement factor over time, we remeasured the intensities with a period duration of the initial 20 s. Comparing the first cycles with 20 s in the beginning with the cycles of 20 s at the end of the measurement, an intensity loss of 25% can be seen. Comparing the intensities at a cycle duration of 2 s with the intensity, which can be measured when going back to the initial cycle duration of 20 s, an intensity loss of 42% can be assigned to the fast period duration. Regarding the reduction at a period duration of 3 s, the last complete reduction can be measured as indicated by the signal intensity touching the baseline. At low cycle durations (5 s and below), interference between the recording speed of the Raman acquisition (~ 500 ms) and the cycle duration of the applied potential starts to occur, as is further described in Chapter 6 in the SI.

Overall, we demonstrated that complete reduction is visible down to period durations of 2 s. Still, the signal intensity decreases while decreasing the time period, which could be crucial, especially for low-emitting small Raman cross-section analytes. Therefore, to overcome this, we chose a period duration of 10 s in our further experiments since this was the lowest tested period duration, not compromising the recorded data in the mentioned way and providing a stable cycling, as evident from Fig. 8.

### Multianalyte measurements

**Fig. 9:**
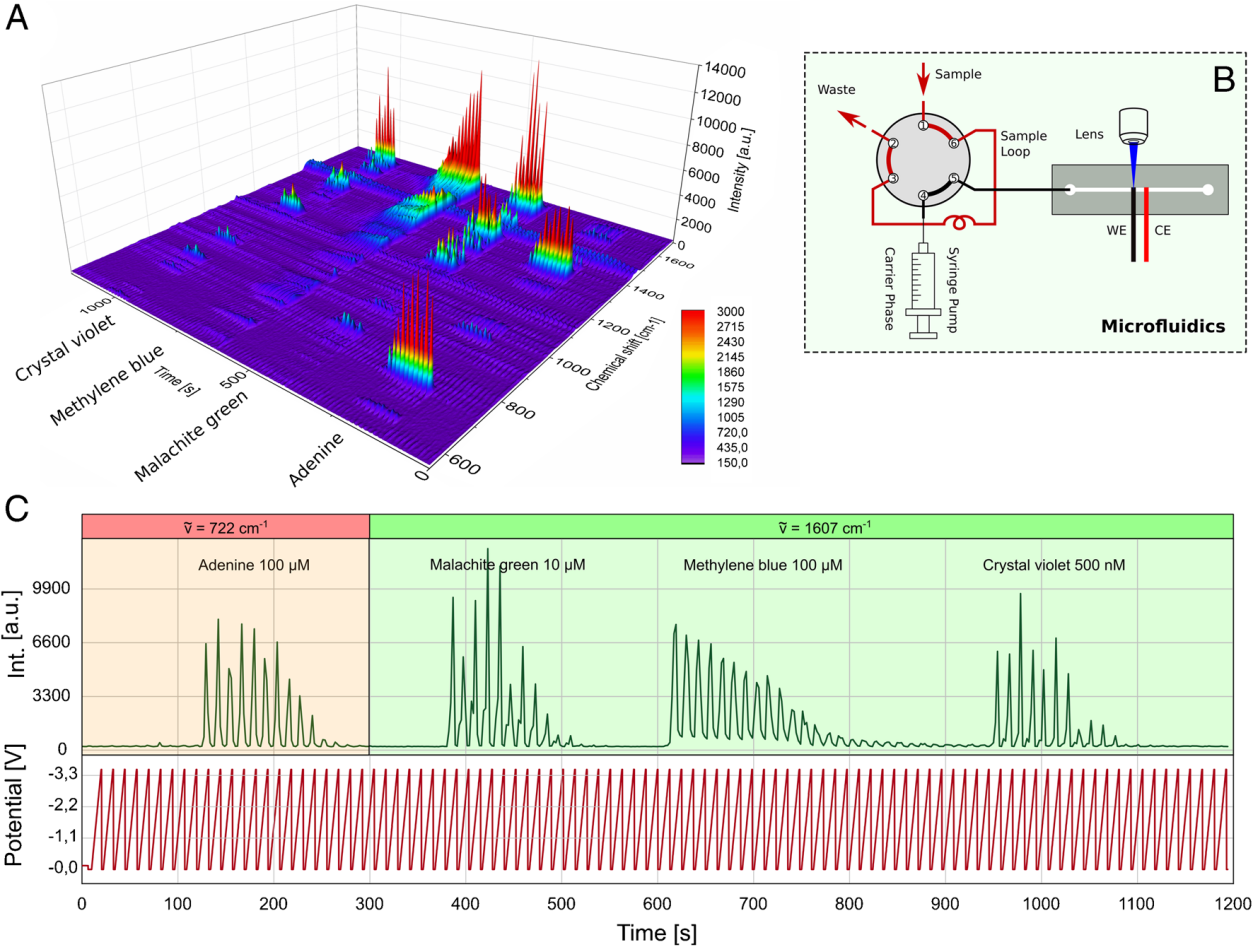
Detection with the following alternating analytes: 100 µM adenine, 10 µM malachite green, 100 µM methylene blue, and 500 nM CV dissolved in dissolved in MeOH/H_2_O 50/50 containing 50 mM Bu_4_NOAc. A potentiostat in 2-electrode configuration as powersource giving a sawtooth potential varying between 0.0 and − 3.5 V and a period duration of 10 s. Measurement parameters: 532 nm; 8.1 mW; 600 lines/mm; objective: 40-fold; integration time: 2 s; flow: 200 µL/min; indicational band: as indicated. In each measurment, 500 µL of sample were sampled via Hexaport ventile. **A** 3D diagram containing the whole Raman intensity curve of the measurement over time. **B** Experimental setup for sampling the analytes containing the Hexaport. WE, working electrode used as SERS substrate. CE, counter electrode. **C** Intensity curve at wavenumber as indicated together with the applied potential. Analytes are indicated for every group of peaks

After evaluating the influence of various experimental parameters on the spectroelectrochemical behaviour of our main model compound, CV, in a flow cell, the final step is to demonstrate the broad applicability of our optimised conditions by sequentially measuring multiple substances. We added a Hexaport valve, as shown in Fig. 9B, to enable multianalyte measurements via flow injection, allowing us to inject CV, malachite green (MG), methylene blue (MB) and adenine (AD) solutions. As previously optimised, we used 50 mM Bu_4_NOAc dissolved in H_2_O/MeOH 50/50. For electrochemical modulation of the SERS signals, we used the previously optimised saw tooth shape potential with a period duration of 10 s and an amplitude of − 3.5 V. We used a potentiostat in the 2-electrode configuration as a power source, which added a small undefined waiting time of 1 s to 2 s after each cycle at 0.0 V. To ensure that all compounds can be measured under the given conditions without adjusting acquisition parameters, we used different concentrations of the SERS dyes. For AD and MB, 100 µM concentrations were used. For MG, we used a concentration of 10 µM. For CV, we had to go down to 500 nM to get signal intensities comparable to those of the other compounds. The Hexaport loop we used had a volume of 500 µL. Considering a 200 µL/min flow rate, the analyte has approximately 150 s to bypass the SERS electrode. Figure 9A displays the resulting time series as a 3D graph. In Fig. 9C, the intensity of the wavenumber of the highest intensity is shown for each compound together with the applied potential. For AD, we used a wavenumber of 722 cm^−1^ as an indicational band. The intensity at the wavenumber of 1607 cm^−1^ was commonly used for all other compounds.

In Fig. 9C, it is visible that the intensity of MB does not touch zero during the 150 s when the analyte continuously flows because the SERS spectrum of the reduced form of MB is visible. Regarding MB a strong tailing is visible. MB is known for its strong memory effect on SERS targets. This tends to limit sample throughput. In the investigated case, the memory effect of MB gradually diminishes with the solvent stream.

In summary, we could measure the spectra of all compounds in one run. The optimised parameters allowed us to perform an online multianalyte measurements. Due to the periodically applied potential, the spectrum of each dye appears to be interrupted and evenly spaced.

### Investigation of non-model compounds

**Fig. 10:**
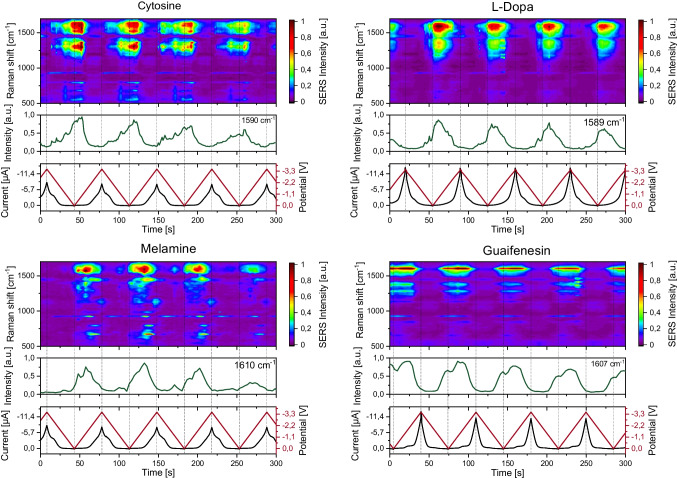
EC-SERS signal progressions of cytosine, L-Dopa, melamine, and guaifenesin. 1 mM concentration and dissolved in 50 mM aqueous Bu_4_NOAc were used. Spectra were recorded under constant flow of 200 µL/min using the 473 nm setup. Power source: frequency generator between 0.0 and − 3.5 V, period duration: 70 s. Parameters: (analyte, laser power, integration time) cytosine, 7.9 mW, 1 s; L-Dopa, 7.9 mW, 1; melamine: 7.9 mW, 3 s; guaifenesin, 7.9 mW, 3 s. 600 lines/mm; objective: 40-fold

So far, we have focused on typical SERS model compounds, particularly CV, to optimise our system and demonstrate multianalyte measurements. In the final step, we aim to determine whether our system, with the optimised parameters, is also suitable for characterising compounds that are not typical SERS model compounds. For these experiments, we used aqueous solutions of 50 mM Bu_4_NOAc as supporting electrolyte. To enable the detection of non-model compounds, we increased their concentrations to 1 mM and, when necessary, adjusted the laser intensity and integration time. We successfully characterised cytosine, L-Dopa, melamine, and guaifenesin. Figure 10 displays the resulting signal progressions alongside the applied potential, current, and the most intense indicative band. While guaifenesin showed a straightforward signal progression, the complex spectroelectrochemical behaviour becomes apparent for the other measured compounds. While the spectroelectrochemical behaviour of melamine [31] and cytosine [32] have been investigated earlier, this is the first EC-SERS study of guaifenesin and L-Dopa.

## Conclusion

Our study demonstrates a simplistic and easy-to-manufacture system for integrating spectroelectrochemical methods with online methods for additional signal enhancement and signal alteration. We optimised and evaluated the usability of parameters such as electrode materials, SERS substrate materials, supporting electrolytes, solvents, and potential curves to maintain a simple system on the one hand and achieve a universal and fast spectroelectrochemical characterisation of SERS analytes on the other hand. We demonstrated the usability of our system for various compounds including SERS model compounds, contaminants, and pharmaceuticals and demonstrated its usability in long-term multicomponent analysis.

Future research should prioritise integrating EC-SERS with analytical online applications, such as HPLC, into further miniaturised, pressure-stable sensors. This should be accompanied by the development of more advanced SERS substrates to enhance signal intensities. The methods and parameters should be optimised to maximise benefits in terms of detection times, signal intensities, and spectroelectrochemical information about the individual compounds of interest. This work serves as a valuable guideline for method development and helps overcome the barriers to seamlessly coupling SERS with high-throughput online methods like HPLC, thereby bringing it closer to routine analytical applications.

## Supplementary Information

Below is the link to the electronic supplementary material.

Supplementary file1 (PDF 2.28 MB)
