# Supplementary material for: Requirements for fast multianalyte detection and characterisation via electrochemical-assisted SERS in a reusable and easily manufactured flow cell
Source: Anal Bioanal Chem. 2025 Feb 3;417(9):1847–61. doi: 10.1007/s00216-025-05763-w (PMC11914304; doi:10.1007/s00216-025-05763-w)
Supplement: Supplementary file 1 — Supplementary file1 (PDF 2.28 MB) [file 216_2025_5763_MOESM1_ESM.pdf]

Supporting Information

Requirements for fast multianalyte detection and characterisation via electrochemical-assisted SERS in a reusable and easily manufactured flow cell

Maximilian E. Blaha[a], Anish Das[a] and Detlev Belder[a*]

[a] Institute for Analytical Chemistry, Leipzig University, Linnéstraße 3, 04103 Leipzig, Germany.

***Corresponding Author:** Detlev Belder, Institute for Analytical Chemistry, Leipzig University, Linnéstraße 3, 04103 Leipzig, Germany. E-mail address: belder@uni-leipzig.de

1. Protocols for SERS substrate manufacturing

This section provides a detailed description of the protocols used for manufacturing the SERS (Surface-Enhanced Raman Spectroscopy) substrates. Characterization of these substrates was conducted using Scanning Electron Microscopy (SEM) and Energy Dispersive X-ray (EDX) analysis. SEM images were captured using two different setups: an FEI Nova NanoLab 200, which is equipped with an additional EDX detector, and a Phenom XL G2 from Thermo Fisher. For EDX measurements, a Bruker Quantax 200 system was utilized to analyze the elemental composition of the substrates. The enhancement factors for the SERS substrates were determined using a 473 nm laser setup, as described in the main article. It is important to note that the protocols used for manufacturing the SERS substrates were not optimized for maximizing the enhancement factor, meaning the enhancement achieved may not represent the maximum potential of the substrates.

1.1 Silver etched with HNO_3

SERS substrate, as used in our previous publication [1], adapted from the literature. [2].

1. Silver wire (0,25 mm; Sigma Aldrich; Germany) was flattened using a mechanical press (PO10H; Paul Otto Weber; Germany) for 120 min at 30 kN.
2. The Silver wire was ultrasonicated for 3 min in methanol (HiPerSolv Chromanorm; VWR; Germany) for cleaning.
3. Etching for 30s in 35 % NH_4OH (Fisher Chemical; Germany)
4. Etching in 6M HNO_3 for 10s.

After etching, we checked if the silver was roughened using a microscope. If no roughening was achieved, we repeated step 4 a second time.

We recorded SEM images, as shown in S1.

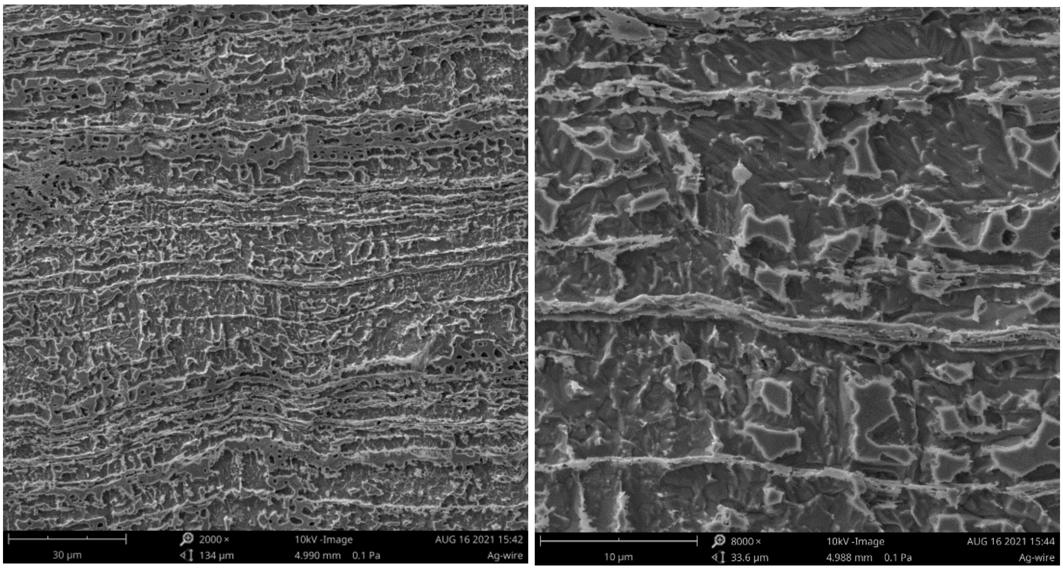

Figure S1: SEM of roughened Silver wire. Device: Phenom XL G2. 10 kV, Detector: ETD-SE. Magnisifications: 2000x and 8000x.

With this protocol, we achieved average enhancement factors of 8.4 x 10^3. Exceptional, the enhancement factors increased to 6.39 x 10^5 using 10 µM CV dissolved in PBS. Using 50 mM Bu_4NOAc in H_2O as an additive in combination with electrochemical enhancement, we achieved enhancement factors of up to 2.65 x 10^6 with an average SERS substrate.

1.2 Copper-based SERS substrate

Here, we pressed and etched copper in a way similar to the method used with the silver wire in the previous approach.

1. Copper wire (0.25 mm; Alfa Aesar; Germany) was flattened using a mechanical press (PO10H; Paul Otto Weber; Germany) for 120 min at 30 kN.
2. The Copper wire was ultrasonicated for 3 min in methanol (HiPerSolv Chromanorm; VWR; Germany) for cleaning.
3. Etching for 30s in 35 % NH_4OH (Fisher Chemical; Germany)
4. Etching in 65% HNO_3 for 10s.

Figure S2 shows SEM-images of Cu-wires after etching.

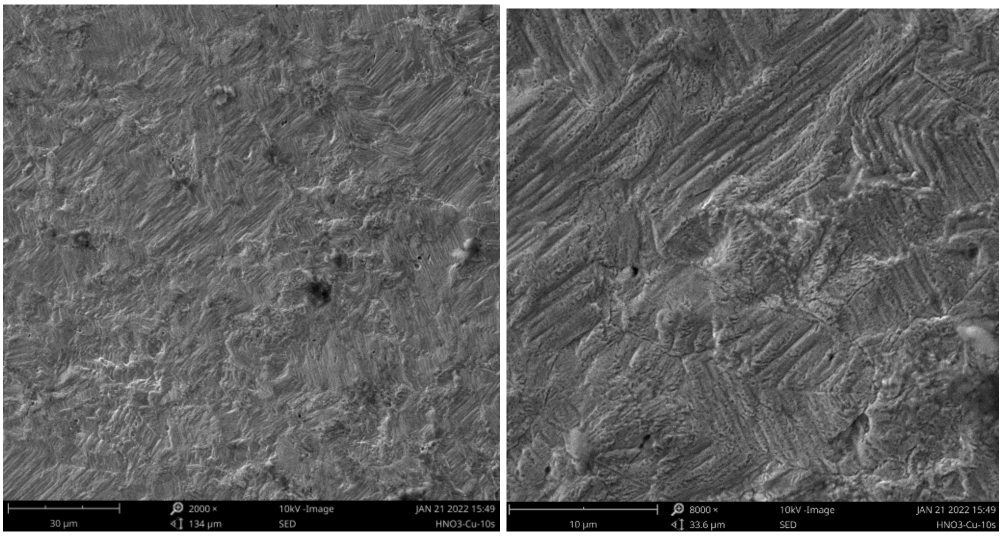

Figure S2: SEM of roughened copper wire after etching. Device: Phenom XL G2, 10 kV, Detector: ETD-SE. Magnifications: 2000x and 8000x.

The resulting SERS substrate produced only weak and unreliable signals, so we added an additional step involving electrical deposition. The chip, which included the etched copper wire and a platinum counter electrode, was flushed with a 10 mM solution of $Cu(OAc)_2$ (p.a.) dissolved in deionized water at a flow rate of 200 µL/min. A voltage of 2.7 V was applied for 2 minutes.

The resulting SERS substrate gave us an enhancement factor of up to 3.5 x 10^3 combining 50 mM Bu_4NOAc in 50/50 H_2O/MeOH as an additive with electrochemical enhancement. Note that the SERS substrate was not optimized. Furthermore, we chose 473 nm for SERS measurements. Copper might perform better in higher wavelengths.

After experiments, the SERS substrate was extracted from the chip after dissolving the glue with acetone overnight. The SEM-images are shown in Figure S3.

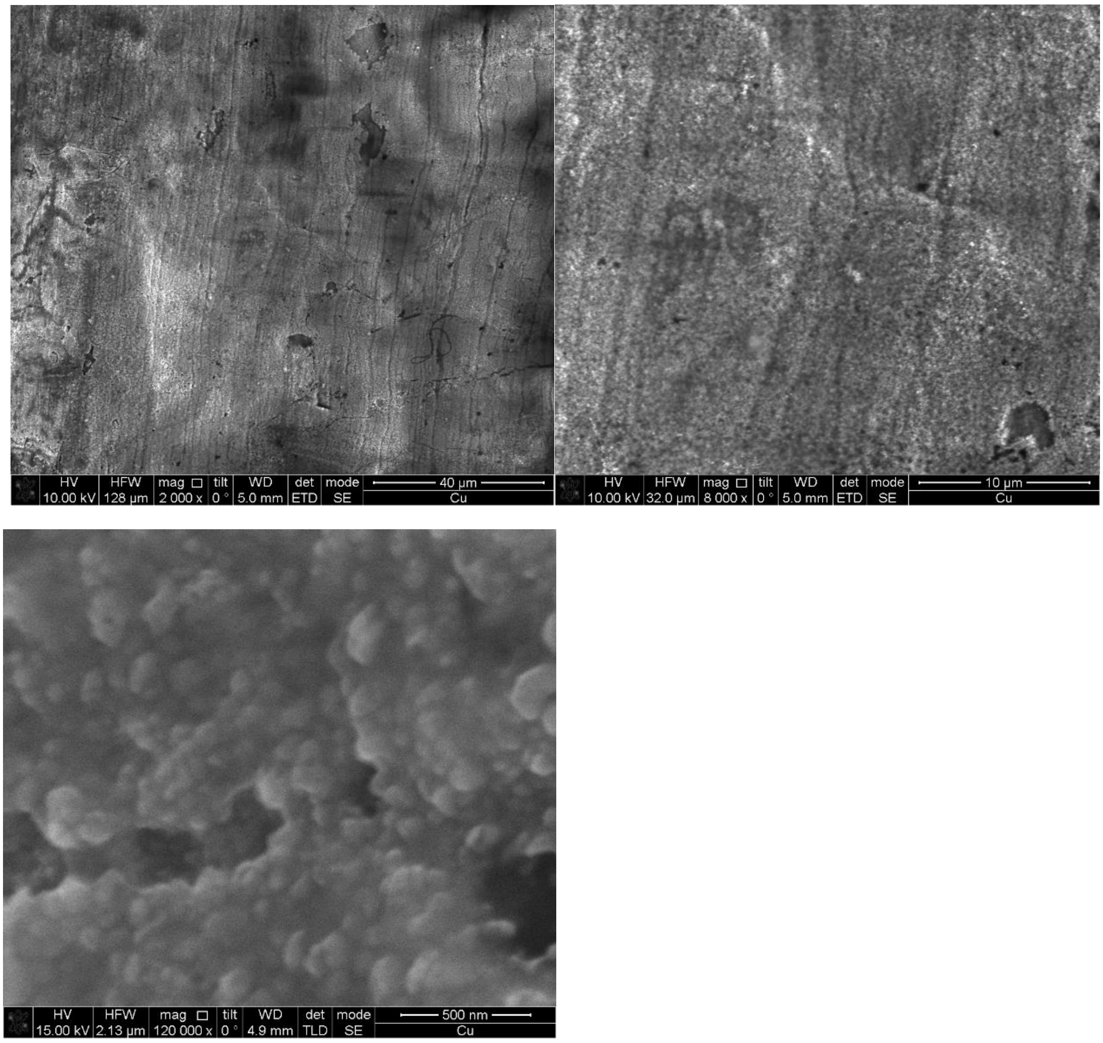

Figure S3: SEM-images of roughened copper wires after etching and electrical deposition of Cu. Device: FEI Nova NanoLab 200. Parameters for 2000x and 8000x images: 10 kV, Detector: ETD-SE. Parameters for 120 000x image: 15 kV, Detector: TLD-SE.

1.3 Gold-based SERS substrate

Gold was pressed and etched as follows.

1. Gold wire (0.25 mm; Alfa Aesar; Germany) was flattened using a mechanical press (PO10H; Paul Otto Weber; Germany) for 120 min at 30 kN.
2. Etching for 30 min Aqua Regia (37% HCl (p.a.) and 65 % HNO3 (p.a.) (4/1) (V/V)) at room temperature.

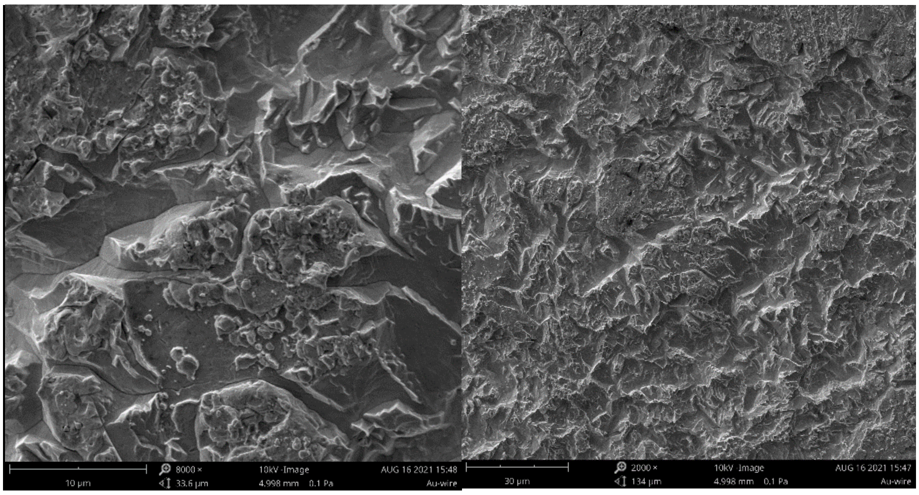

Figure S4: SEM of gold wire after etching. Device: Phenom XL G2, 10 kV, Detector: ETD-SE. Magnifications: 2000x and 8000x.

The resulting SERS substrate gave us only weak and unreliable SERS signals, so we included an additional Step of electrical deposition. The chip, including the etched Au wire and a Pt-counter electrode, was flushed with 10 mM $HAuCl_4$ (Sigma Aldrich, Germany) in d.i. Water at a rate of 200 µL/min. A voltage of -2.7 V was applied over 1 min.

The resulting SERS substrate gave us an enhancement factor of up to 1.0 x 10^3 using 10 µM CV and 50 mM Bu_4NOAc. The substrate showed an overall poor enhancement. Using higher wavelengths instead of 473 nm might give better results. However, the enhancement is lost fast during exposure and the application of electrochemical potentials.

After the experiments, the SERS substrate was removed from the chip by dissolving the adhesive in acetone overnight. The SEM-images of the substrate are shown in Figure S5.

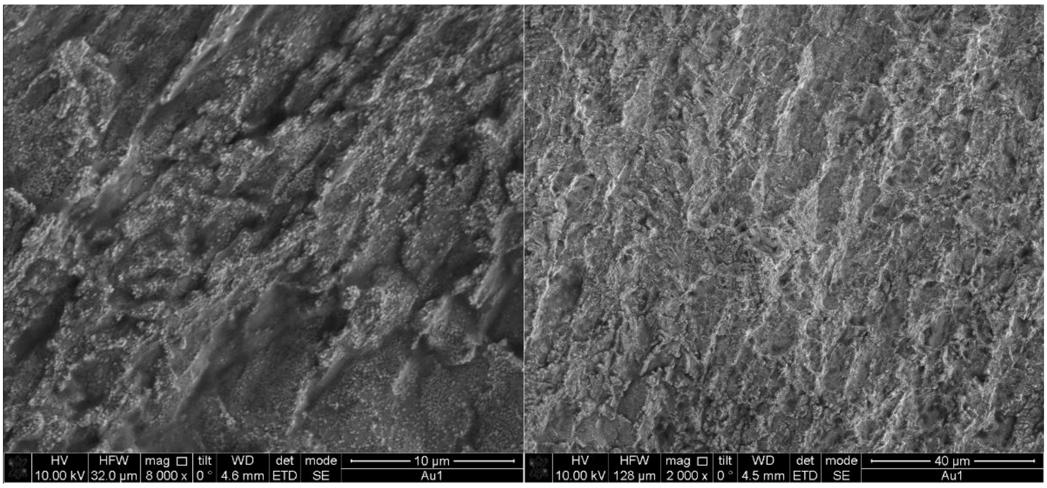

Figure S5: SEM-images of roughened copper wires after etching and electrical deposition of Au. Device: FEI Nova NanoLab 200. Parameters for 2000x and 8000x images: 10 kV, Detector: ETD-SE.

1.4 $Au_{31}Ag_{69}$-based SERS substrate I_2/KI etching

The SERS substrate was manufactured as follows:

1. Gold/Silver-wire ($Au_{31}Ag_{69}$, 0.25 mm; HMW Hauner GmbH & Co. KG, Germany) was flattened using a mechanical press (PO10H; Paul Otto Weber; Germany) for 120 min at 30 kN.
2. For cleaning, the wire was ultrasonicated for 3 min in methanol (HiPerSolv Chromanorm; VWR; Germany).
3. The wire was etched using a solution of 1 g I_2 (p.a.), 4 g KI (p.a.) and 40 ml H_2O (d.i.) for 20 minutes.

After etching, SEM images were recorded, as shown in Figure S6. EDX analysis revealed that the surface was covered with AgI.

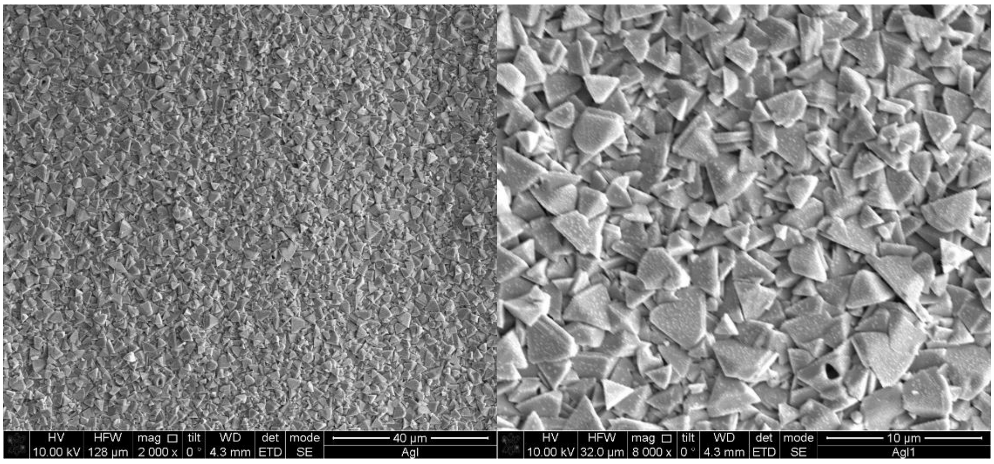

Figure S6: SEM-images of roughened $Au_{31}Ag_{69}$ wire. The surface is coated with AgI crystals. Device: FEI Nova NanoLab 200. Parameters for 2000x and 8000x images: 10 kV, Detector: ETD-SE.

The resulting SERS substrate showed an enhancement factor of 1.8 x 10^4 using 10 µM CV dissolved in 50/50 MeOH/H_2O and 50 mM Bu_4NOAc as an additive combined with electrochemical enhancement. We also tried to etch the wire for 10 min, which gave practically no SERS signal. To achieve a stable SERS signal, it was necessary to perform multiple reduction cycles to remove AgI from the surface.

1.5 $Au_{31}Ag_{69}$-based SERS substrate HNO_3 etching

The SERS substrate was manufactured as follows:

1. Gold/Silver-wire ($Au_{31}Ag_{69}$, 0.25 mm; HMW Hauner GmbH & Co. KG, Germany) was flattened using a mechanical press (PO10H; Paul Otto Weber; Germany) for 120 min at 30 kN.
2. For cleaning, the wire was ultrasonicated for 3 min in methanol (HiPerSolv Chromanorm; VWR; Germany).
3. The wire was etched using 65 % HNO_3 for 120 minutes.

Figure S7 shows SEM-images after roughening. The left side shows the structure on the bare surface. The right side shows an image of the broken edge of the substrate. EDX measurements gave an elementary composition of $Au_{75}Ag_{25}$ after etching.

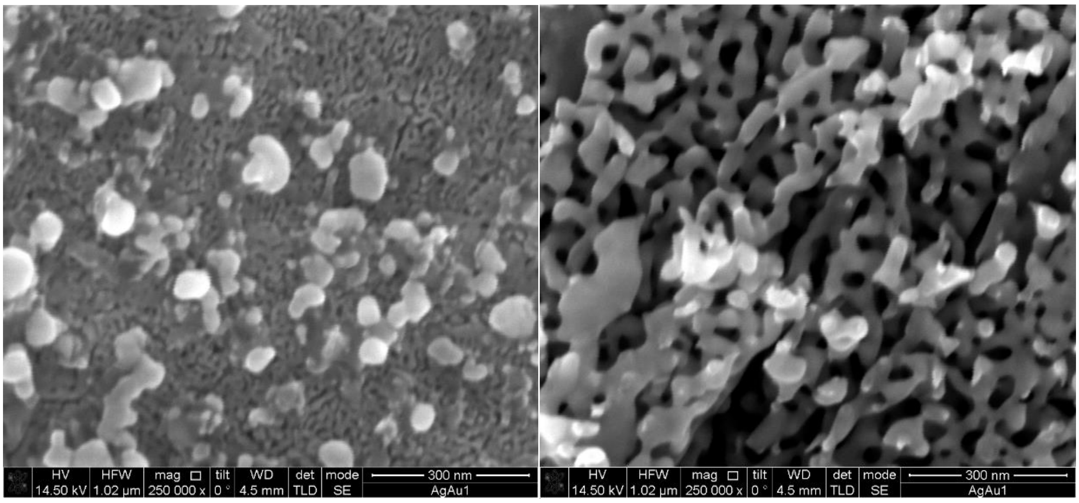

Figure S7: SEM images of an edged $Au_{31}Ag_{69}$ target. Left: Image of the blank surface. Right: Image of the broken edge of the substrate. Magnisifications: 250 000 x; Parameters 14,5 kV, TLD-SE

The resulting SERS substrate showed an enhancement factor of 7.3 x 10^3 using 10 µM CV dissolved in PBS. After one reduction cycle, the signal did not regrow, making this substrate poorly usable for our application. We repeated the measurements with an 633 nm laser which is more suitable for Au-SERS targets with similar results.

Regarding the etching protocols, 60 min etching time in HNO_3 and below did not enhance Raman-Signals. Etching times of 180 min and above resulted in a substrate too fragile to be included in our system.

2. Dataprocessing

Each Raman spectrum was smoothed and had its background corrected. Background correction is essential because the background signal also fluctuates depending on the applied potential. Background subtraction enables the differentiation between fluctuations in background intensity and changes in the actual Raman signal intensity.

3. Conductivity and pH of aqueous electrolyte solutions

In our experiments, we tried different aqueous electrolyte solutions. The related pH and conductivity are noted. In the experiments, the influence of the electrolyte on the EC-SERS behaviour was investigated. When the conductivity of the solution is too low, electrochemical processes might become incomplete. Table S1 contains the conductivity and pH related to the investigated supporting electrolytes.

Table S1: Conductivities and pH levels of every electrolyte solution used at ambient temperature.

Supporting electrolyte	Concentration [mM]	pH	Conductivity [mS/cm]
PBS	66	7.01	8.19
NH_4OAc	100	6.65	9.08
NaOAc	100	7.88	6.34
KNO_3	100	5.54	11.92
Bu_4NOAc	100	4.66	3.51
NaCl	100	5.60	10.56
Bu_4NHSO_4 + NaOH	100	4.70	13.22
NaCitrate	100	9.05	17.28

The related intensity curves are displayed in Figure S8.

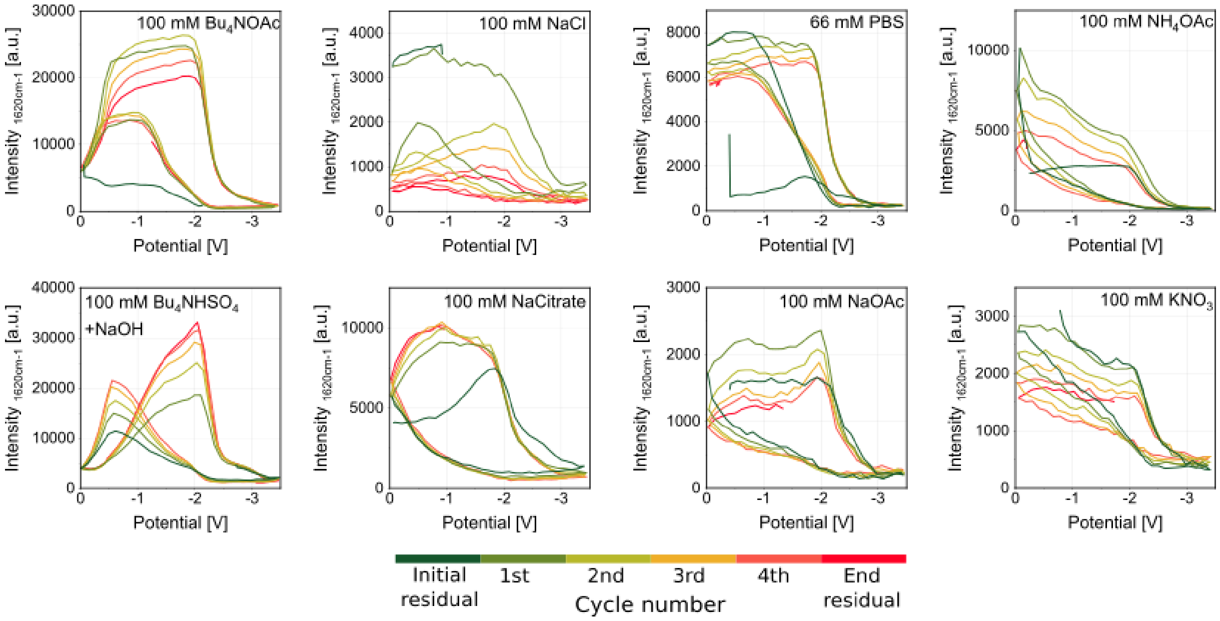

Figure S8: Intensity curve of 10 µM CV dissolved in aqueouse electrolyte solution containing different supporting electrolytes with the indicated concentration. Power source: Frequency generator with triangular potential between 0.0 V and -3.5 V and a period duration of 70 s. Measurement parameters: 473 nm; 2.5 mW; 600 lines/mm; objective: 40-fold; integration time: 1 s; flow: 200 µL/min; indicational band: as indicated)

The intensity curves vary significantly depending on the supporting electrolyte used, both in terms of shape and the maximum intensities achieved. Bu_4NOAc, Bu_4NHSO_4, PBS, and NaCitrate have already been discussed in the main paper. Notably, when NH_4OAc and KNO_3 were used as supporting electrolytes, the intensity began to diminish even before the expected onset of the reduction. In contrast, NaOAc exhibited an increase in intensity just before the signal declined. We believe these differences can be attributed to variations in the structure of the electrochemical double layer, which influences the adsorption of crystal violet (CV) onto the surface of our roughened Ag-SERS substrate. To precisely investigate these effects, it would be necessary to conduct referenced measurements using a potentiostat and a three-electrode system.

A gradual decrease in signal intensity was observed when NaCl was used as the electrolyte. Changing the position on the Ag-SERS substrate temporarily restored the signal intensity, but it quickly diminished again. This suggests that concentrated halide solutions are unsuitable for use with Ag-SERS substrates during electrochemical applications. We believe that a light reaction between silver and the halides at the excitation point leads to a loss of plasmonic activity in the Ag substrate. When using Bu_4NBr and Et_4NI, we observed an even more rapid signal loss, rendering these electrolytes unusable in our setup.

Other electrolytes, such as $Na_2S_2O_3$ (meant to keep the silver surface free from oxides), $Na_2B_4O_7$, and $B(OH)_3$ (intended to mimic sodium borate buffers often used in HPLC), were also tested. However, these electrolytes proved incompatible with our model analyte, crystal violet (CV), as evidenced by a gradual decolorization of the solution.

We further conclude that the supporting electrolyte may interfere with the adsorption of the analyte on the silver surface, depending on the applied potential, the specific ion mixture in the solution, and the stability of potential analyte-electrolyte complexes. We believe these interactions vary between different analytes, necessitating individual optimizations. A thorough understanding of the kinetics and structure of the electrochemical double layer would require a spectroelectrochemical investigation, which falls outside the primary scope of our study.

4. Diagrams, conductivity and pH of varying concentrations of Bu_4NOAc

To evaluate the effect of varying Bu_4NOAc concentrations on the spectroelectrochemical behaviour of our system, we gradually reduced its concentration in aqueous solutions. A test solution containing 10 µM crystal violet (CV) was flowed through the chip at a rate of 200 µL/min. A triangular potential ranging from 0.0 V to -3.5 V was applied over a 70-second cycle, repeated multiple times. Detection was performed using a laser with a power of 2.5 mW (473 nm) and an integration time of 1 second. For the measurement with the solution containing 50 mM Bu_4NOAc, we reduced the laser power to 0.25 mW to ensure the detector could accurately capture all incoming photons, with the measured intensities normalized 10-fold accordingly. In the subsequent graphs, the intensity of crystal violet at a wavenumber of 1620 cm^{-1} is plotted on the y-axis, and the applied potential on the x-axis. A full reduction of our model analyte was achieved with Bu_4NOAc concentrations as low as 10 mM. At concentrations below 10 mM, the solution's conductivity was insufficient, resulting in a residual signal at -3.5 V.

Table S2: Conductivities and pH levels of different concentrations of Bu_4NOAc in aqueous solution at ambient temperature.

Concentration of Bu_4NOAc [mM]	pH	Conductivity [mS/cm]
100	4.62	3.51
50	4.64	1.96
10	4.70	0.41
5	4.74	0.23
1	4.77	0.08

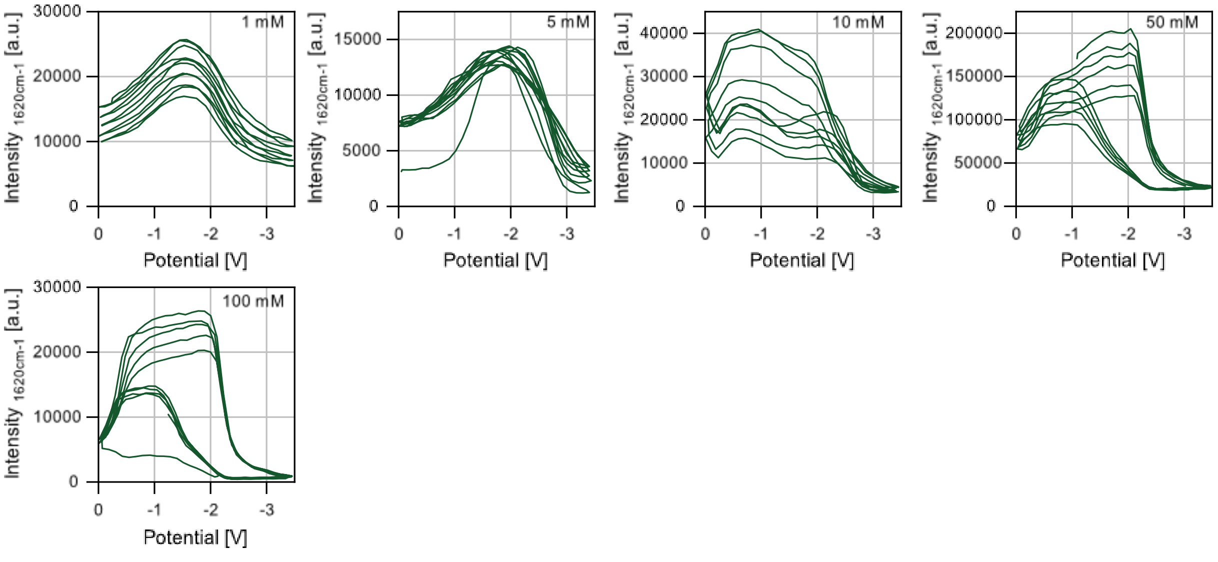

Figure S9: Intensity curve of 10 µM CV dissolved water containing different concentrations of Bu_4NOAc. Power source: Frequency generator with triangular potential between 0.0 V and -3.5 V and a period duration of 70 s. Measurement parameters: 473 nm; 2.5 mW; 600lines/mm; objective: 40-fold; integration time: 1 s; flow: 200 µL/min; indicational band: as indicated. At 50 mM concentration, a laser power of 0.25 mW is used to avoid excess of measurable photons at the detector. The intensity was corrected by multiplying 10 fold accordingly.

5. Diagrams and conductivity of varying solvent mixtures

To investigate the effect of different solvent mixtures on our system, we began with aqueous solutions and progressively increased the proportions of MeOH and MeCN. We utilized our described chip, flushed it with solvent mixtures containing 10 µM crystal violet (CV) and 50 mM Bu_4NOAc, and applied a triangular potential ranging from 0.0 to -3.5 V with a duration of 70 seconds per cycle. For solvent mixtures containing 60% MeCN and higher, we increased the negative potential while maintaining the 70-second cycle duration. Measurements were conducted using our 532 nm setup, as described in the

main paper. To evaluate performance, we plotted the intensity of CV at a wavenumber of 1607 cm^{-1} as a function of the applied potential. Note that intensities should be compared with caution, as each measurement was taken from a different spot on the SERS substrate. The corresponding diagrams are shown in Figure S10.

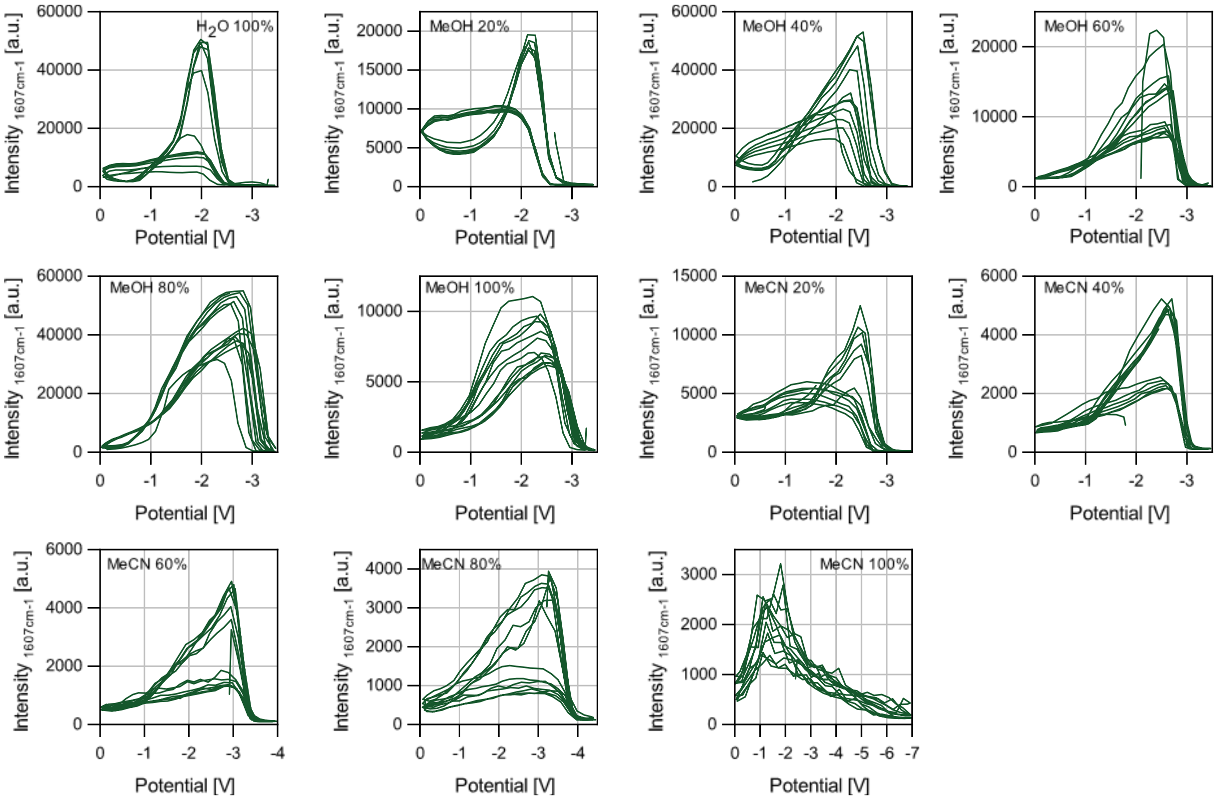

Figure S10: Intensity curve of 10 µM CV dissolved in different solvent mixtures containing 50 mM Bu_4NOAc. Power source: Frequency generator with triangular potential between 0.0 V and -3.5 V and a period duration of 70 s. Measurement parameters: 532 nm; 4.1 mW; 600lines/mm; objective: 40-fold; integration time: 1 s; flow: 200 µL/min; indicational band: as indicated).

For every used solvent mixture, we measured the conductivity. Results are noted in Table S3.

Table S3: Conductivities of every used solvent mixture containing 50 mM Bu_4NOAc.

Solvent Mix	Conductivity [mS/cm]
H_2O 100%	1.73
H_2O 80%/MeOH 20%	1.28
H_2O 60%/MeOH 40%	1.12
H_2O 50%/MeOH 50%	1.10
H_2O 40%/MeOH 60%	1.13
H_2O 20%/MeOH 80%	1.33
MeOH 100%	1.83
H_2O 80%/MeCN 20%	1.65
H_2O 60%/MeCN 40%	1.81
H_2O 50%/MeCN 50%	1.91
H_2O 40%/MeCN 60%	2.02
H_2O 20%/MeCN 80%	2.35
MeCN 100%	2.00

6. A note about the interference of acquisition and the potential curve

When measuring with long period durations and an acquisition time of around 500 ms, each spectrum is fully captured during one of the distinct phases: depolarization, signal enhancement, and reduction.

However, with shorter period durations, some of these phases may overlap during spectrum acquisition, causing differences in intensity to be averaged out. This overlap is responsible for several phenomena observed in the intensity curves at short period durations: 1) The intensity during reduction does not drop to zero, 2) The maximum achieved intensity fluctuates significantly, and 3) Overall intensity levels are lower. Additionally, at period durations of 2 s and 3 s, the signal exhibits periodic fluctuations. This behavior is attributed to the interaction between the periodicity of the applied potential and the periodicity of the spectrum measurement, resulting in a beat pattern.

7. Investigation of secondary analytes

As discussed in the main-paper we carried out multianalyte measurements of malachite green, adenine and methylene blue besides the crystal violet (CV) already investigated. In order to get a better understanding of their SERS behaviour under the influence of potentials we investigated these analytes independently with the parameters already established for CV. For methylene blue we had to use MeCN as solvent in order to fully remove it from the Ag-surface of the SERS substrate. The Intensity curves for each analyte are displayed in Figure S11.

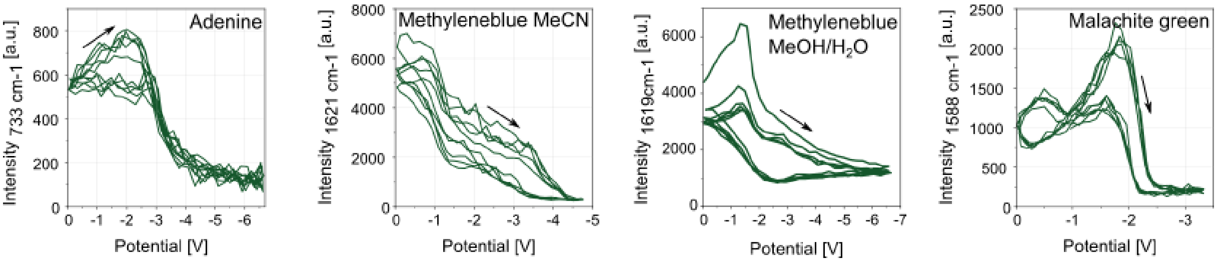

Figure S11: Intensity curves of adenine, methylene blue and malachitegreen. Measurement parameters: 473 nm; 2.5 mW; 600lines/mm; objective: 40-fold; integration time: 1 s; flow: 200 µL/min; indicational band: as indicated. Power source: Frequency generator with triangular potential and a period duration of 70 s. 50 mM Bu_4NOAc in the used solvent accordingly: adenine: 100 µM 50/50 $MeOH/H_2O$; methylene blue 100 µM: MeCN; methylene blue 100 µM: 50/50 $MeOH/H_2O$; malachite green 10 µM: 50/50 $MeOH/H_2O$.

8. Multianalyte measurements

In the main article we discuss the multianalyte measurement. In Figure S12, the extracted SERS spectra of each compound investigated in the multianalyte measurement are shown. We displayed the signals of the first three cycles for each compound. For each displayed spectrum, we alssigned the potential and time accordingly. Black coloured are the spectra with the lowest indicational intensity for each analyte. The red spectrum shows the spectrum with the highest intensity for each cycle. And blue indicates the point when the Voltage changes from -3.5 V to 0.0 V. When no blue line is indicated, as for malachite green, methylene blue and crystal violet the spectrum with the minimal intensity falls together with the spectrum at the point when the applied potential switches from -3.5 to 0.0 V and both together are indicated in black colour.

For adenine, the lowest intensity was observed at potentials between -1.41 and -0.44 V, while the highest intensity occurred at potentials between -2.48 and -2.87 V. Adenine, a base in an acidic solution, forms an adenine-cation. We believe that the negative potentials attract this cation, thereby enhancing the recorded signal.

For malachite green and crystal violet, the maximum intensities were observed between -2.33 V and -1.70 V for malachite green, and between -2.58 V and -2.30 V for crystal violet. The minimum intensity, indicating the absence of Raman-active compounds, was reached between -3.15 V and the point where the potential switches from -3.5 V to 0.0 V.

For methylene blue, the highest intensities were achieved between -1.29 V and -2.73 V, with the lowest intensity occurring at the switch point between -3.5 V and 0.0 V. At this lowest intensity point, a residual spectrum with a broad band around 1600 cm^{-1} is visible which conclude to be the spectrum of the

reduced form of methylene blue. The other compounds did not exhibit a significant spectrum of their reduced forms that was clearly distinguishable from the background.

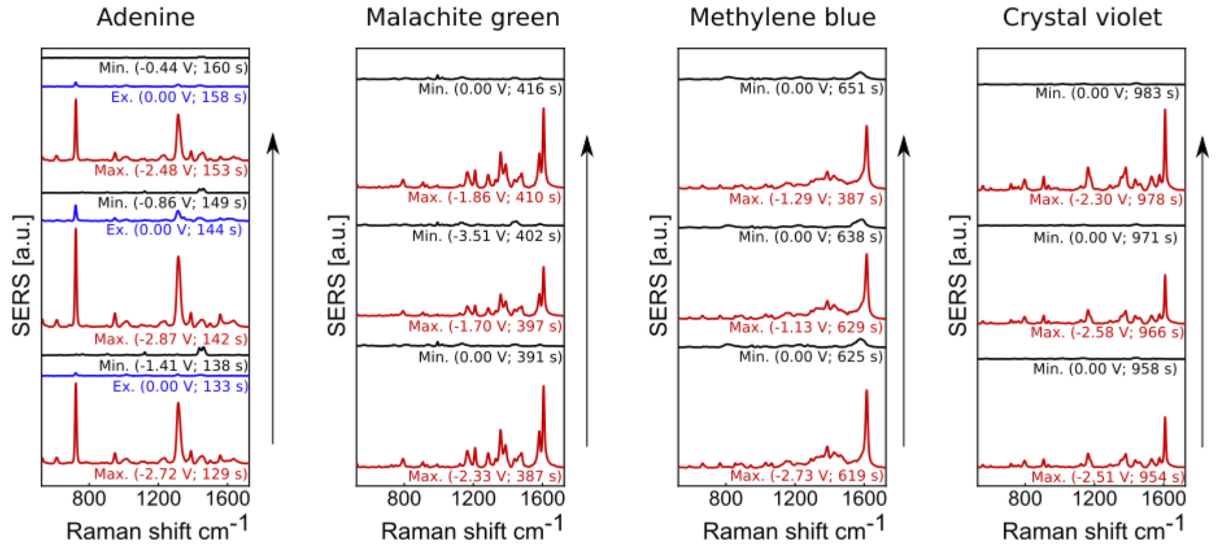

Figure S12: SERS-spectra as obtained from multianalyte measurements as displayed and discussed in the main paper Figure 9. 473 nm; 2.5 mW; 600lines/mm; objective: 40-fold; integration time: 1 s; flow: 200 µL/min SERS spectra of adenine at maximum Intensity (red), the lowest intensity (black) and at the point when the potential switches from -3.5 V to 0.0 V (blue). Included are the first 3 cycles of detection and reduction. malachite green, methylene blue and crystal violet same as for adenine. Point of minimum intensity is the same as the point when the potential switches from -3.5 V to 0.0 V.

9. Influence of reductive potentials on the memory effect of crystal violet.

Crystal violet (CV) exhibits a strong memory effect on SERS substrates. The memory effect refers to the slow desorption or permanent adsorption of the analyte to the SERS substrate, causing carryover effects that result in mixed spectra when multiple analytes are analyzed one after another. [3–5] In general, it is possible to remove analytes from the SERS substrate by applying a potential. In the following experiment, we investigated whether the conditions explored in this study could effectively strip CV from the electrode and accelerate its otherwise diffusion-controlled desorption.

For this experiment, we used our 473 nm setup. In the described flow cell, an aqueous solution of 10 µM CV in 66 mM PBS (pH 7.00) was pumped through the chip via syringe pumps (Nemesys, Cetoni, Korbussen, Germany) for the first 60 s. Following this, a buffer solution without CV was pumped through the chip. After a waiting period of 60 s (total time of 120 s), a potential of -4 V was applied in one trial, while in another trial, no potential was applied. After an additional 60 s, the potential was disabled again.

Figure S13 shows the signal profiles of both experiments at a representative wavenumber of 1618 cm^{-1}. The black curve represents the intensity profile with applied potential, while the red curve shows the profile without applied potential. Once the pure buffer starts flowing through the chip, both curves exhibit an approximately exponential decay in the signal intensity of CV. When the potential is applied in the black curve, the signal intensity rapidly drops to the background noise level. However, once the potential is switched off, the signal intensity partially returns.

We conclude that CV^+ is reduced to a form ($CV^•$) that is barely detectable by SERS. This reduced form remains partially adsorbed on the SERS substrate. When the potential is removed, the adsorbed reduced $CV^•$ is re-oxidized, making it visible to SERS once again. Our findings indicate that the applied conditions do not fully desorb the CV from the SERS substrate. Other electrochemical conditions might allow for a reductive (or oxdative) desorption. [6–9]

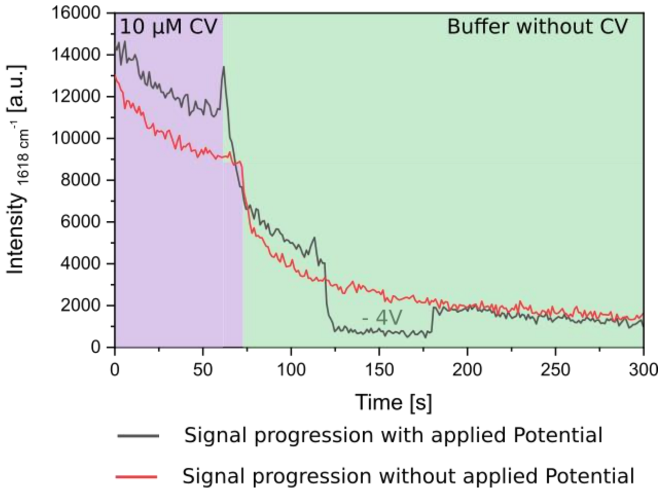

Figure S13: Signal progression of crystal violet (CV) over time. CV was dissolved in 66 mM PBS, pH 7.00, using a 473 nm wavelength; laser power: 2.5 mW; grating: 600 lines/mm; objective: 40× magnification; integration time: 1 s; flow rate: 200 µL/min. From 0 s to 60 s, both curves show 10 µM CV dissolved in buffer flushed through the chip. From 60 s to 120 s, buffer without dissolved CV is flushed through the chip for both curves. From 120 s to 180 s, a potential of -4 V is applied in the black curve, while no potential is applied to the red curve. From 180 s to 300 s, no potential is applied in either curve.

10. Comparison of the microstructure of the SERS-substrates before and after measurements

One potential reason for changes in signal intensity over time could be alterations in the SERS substrate's microstructure during electrochemical cycling. To rule out this possibility, we manufactured an Ag-SERS target using pressed and polished Ag wire (Lapping Film 261x, 3M, USA) and wet-etched it as described in Chapter 1.1. We then compared the microstructure before and after electrochemical cycling. For the experimental settings, we used our initial unoptimized parameters: a 200 µL/min flow of a 10 µM crystal violet solution dissolved in 66 mM PBS (pH 7.00). Electrochemical cycling was carried out between 0.0 V and -3.5 V, with a period duration of 70 seconds over a total of 5 minutes. We employed our 473 nm setup with a laser power of 2.5 mW. To remove the electrode from the chip post-measurement, the chip was carefully broken with a hammer, and the electrodes were detached using a scalpel. The electrodes were then cleaned with acetone and dried under a nitrogen stream. Electron microscopy images, displayed in Figure S14, compare the nanostructure of the SERS substrate before and after electrochemical cycling. During comparative imaging, we captured images from the exact same spot on the SERS substrate both before and after cycling, indicating that surface changes were minimal. We minor surface could also be attributed to slight focus adjustments in the SEM or to the removal process itself proving that surface changes are not responsible for changes in signal intensity of our analyte.

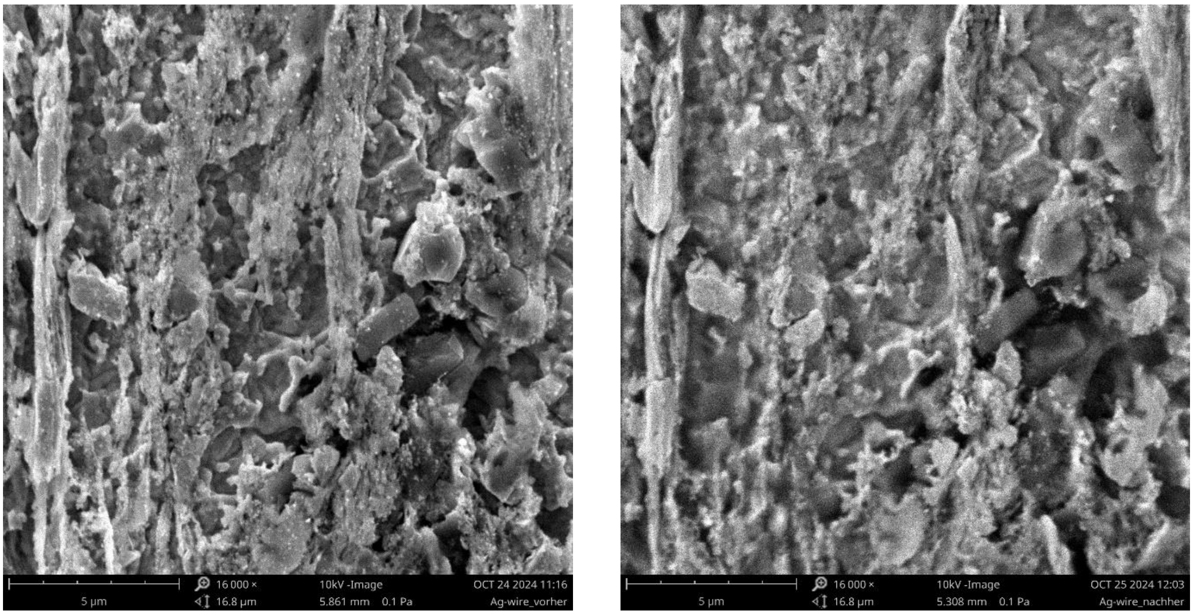

Figure S14: Comparative SEM of a wet etched Ag-SERS-substrate before (left) and after (right) use. Phenom XL G2, 10 kV, Detector: ETD-SE. Magnifications: 16 000 x.

References

1. Höhn E-M, Panneerselvam R, Das A, Belder D. Raman Spectroscopic Detection in Continuous Microflow Using a Chip-Integrated Silver Electrode as an Electrically Regenerable Surface-Enhanced Raman Spectroscopy Substrate. Anal Chem. 2019; https://doi.org/10.1021/acs.analchem.9b01514
2. Wijesuriya S, Burugapalli K, Mackay R, Ajaezi GC, Balachandran W. Chemically Roughened Solid Silver: A Simple, Robust and Broadband SERS Substrate. Sensors (Basel). 2016; https://doi.org/10.3390/s16101742
3. Chen J, Li S, Yao F, Bao F, Ge Y, Zou M, Liang P, Chen Q. Progress of Microfluidics Combined with SERS Technology in the Trace Detection of Harmful Substances. Chemosensors. 2022; https://doi.org/10.3390/chemosensors10110449
4. Cialla D, Hübner U, Schneidewind H, Möller R, Popp J. Probing innovative microfabricated substrates for their reproducible SERS activity. Chemphyschem. 2008; https://doi.org/10.1002/cphc.200700705
5. Markin AV, Arzhanukhina AI, Markina NE, Goryacheva IY. Analytical performance of electrochemical surface-enhanced Raman spectroscopy: A critical review. TrAC Trends in Analytical Chemistry. 2022; https://doi.org/10.1016/j.trac.2022.116776
6. Elgrishi N, Rountree KJ, McCarthy BD, Rountree ES, Eisenhart TT, Dempsey JL. A Practical Beginner’s Guide to Cyclic Voltammetry. J. Chem. Educ. 2018; https://doi.org/10.1021/acs.jchemed.7b00361
7. Arinaga K, Rant U, Knezević J, Pringsheim E, Tornow M, Fujita S, Abstreiter G, Yokoyama N. Controlling the surface density of DNA on gold by electrically induced desorption. Biosensors and Bioelectronics. 2007; https://doi.org/10.1016/j.bios.2007.04.012
8. Sun K, Jiang B, Jiang X. Electrochemical desorption of self-assembled monolayers and its applications in surface chemistry and cell biology. Journal of Electroanalytical Chemistry. 2011; https://doi.org/10.1016/j.jelechem.2010.11.008
9. Widrig CA, Chung C, Porter MD. The electrochemical desorption of n-alkanethiol monolayers from polycrystalline Au and Ag electrodes. Journal of Electroanalytical Chemistry and Interfacial Electrochemistry. 1991; https://doi.org/10.1016/0022-0728(91)85271-P
